# Global stability of secondary DENV infection models with non-specific and strain-specific CTLs

**DOI:** 10.1016/j.heliyon.2024.e25391

**Published:** 2024-01-29

**Authors:** Aeshah A. Raezah, A.M. Elaiw, M.A. Alshaikh

**Affiliations:** a Department of Mathematics, Faculty of Science, King Khalid University, Abha 62529, Saudi Arabia; b Department of Mathematics, Faculty of Science, King Abdulaziz University, P.O. Box 80203, Jeddah 21589, Saudi Arabia; c Department of Mathematics, College of Science, Taif University, P.O. Box 11099, Taif 21944, Saudi Arabia

**Keywords:** 34D20, 34D23, 37N25, 92B05, Dengue virus, Immune response, Global stability, Lyapunov function

## Abstract

Dengue virus (DENV) is a highly perilous virus that is transmitted to humans through mosquito bites and causes dengue fever. Consequently, extensive efforts are being made to develop effective treatments and vaccines. Mathematical modeling plays a significant role in comprehending the dynamics of DENV within a host in the presence of cytotoxic T lymphocytes (CTL) immune response. This study examines two models for secondary DENV infections that elucidate the dynamics of DENV under the influence of two types of CTL responses, namely non-specific and strain-specific responses. The first model encompasses five compartments, which consist of uninfected monocytes, infected monocytes, free DENV particles, non-specific CTLs, and strain-specific CTLs. In the second model, latently infected cells are introduced into the model. We posit that the CTL responsiveness is determined by a combination of self-regulating CTL response and a predator-prey-like CTL response. The model's solutions are verified to be nonnegativity and bounded and the model possesses two equilibrium states: the uninfected equilibrium ${\mathrm{EQ}}^{0}$ and the infected equilibrium ${\mathrm{EQ}}^{⁎}$. Furthermore, we calculate the basic reproduction number $R_{0}$, which determines the existence and stability of the model's equilibria. We examine the global stability by constructing suitable Lyapunov functions. Our analysis reveals that if $R_{0}\leq 1$, then ${\mathrm{EQ}}^{0}$ is globally asymptotically stable (G.A.S), and if $R_{0}>1$, then ${\mathrm{EQ}}^{0}$ is unstable while ${\mathrm{EQ}}^{⁎}$ is G.A.S. To illustrate our findings analytically, we conduct numerical simulations for each model. Additionally, we perform sensitivity analysis to demonstrate how the parameter values of the proposed model impact $R_{0}$ given a set of data. Finally, we discuss the implications of including the CTL immune response and latently infected cells in the secondary DENV infection model. Our study demonstrates that incorporating the CTL immune response and latently infected cells diminishes $R_{0}$ and enhances the system's stability around ${\mathrm{EQ}}^{0}$.

## Highlights

• The study focuses on understanding the dynamics of Dengue virus (DENV) in the presence of cytotoxic T lymphocytes (CTL) immune response.
• Two mathematical models are studied, incorporating non-specific and strain-specific CTL responses.
• The second model incorporates latently infected cells.
• By including CTLs and latently infected cells in the model, stability around the uninfected equilibrium is enhanced.

## 1 Introduction

The Dengue virus (DENV) is an arbovirus transmitted by mosquitoes, particularly Aedes albopictus and Aedes aegypti, leading to the onset of dengue fever. Symptoms encompass severe headache, high fever (40 ^∘^C), rash, muscle and joint pains, nausea, pain behind the eyes, swollen glands, and vomiting [1]. With almost half of the global population at risk, an estimated 100 to 400 million infections occur annually [1], predominantly in tropical and subtropical regions.

DENV targets various cells, including monocytes, macrophages, dendritic cells, endothelial, and epithelial cells [2]. The interplay of innate and adaptive immune responses is crucial in combating DENV [3]. The innate immune response, acting as the initial defense line, involves early activation of Interferon (IFN) and natural killer (NK) cells, contributing to the containment of DENV spread and clearance of infected cells.

The adaptive immune response, albeit slower to activate, ultimately eliminates DENV from the body [4]. B cells and cytotoxic T lymphocytes (CTLs) are integral components of this response. B cells produce antibodies to neutralize DENV particles, while CTLs target and eliminate DENV-infected cells. DENV comprises four distinct serotypes (DENV1-4), each with different genotypes [5].

Generally, infection with one DENV serotype confers protective immunity against that specific serotype but not against others [6]. Primary infections result in lifelong immunity to the original DENV strain [7]. In secondary infections with a different DENV serotype, two CTL immunities are triggered: non-specific CTLs from the primary infection and strain-specific CTLs against the new DENV serotype [7].

### 1.1 Mathematical models for DENV infection

Mathematical modeling serves as a valuable approach in comprehending DENV infection dynamics, offering a cost-effective alternative to experimental assessments. This method enables the assessment of interactions between viruses, host cells, and immune cells, providing insights into the intricate dynamics of viral infections.

#### 1.1.1 Primary DENV infection models

Models pertaining to primary DENV infection within the host have been developed in recent years, with various mathematical formulations available in the literature (refer, for instance, [4], [6], [7], [8], [9], [10], [11], [12], [13], [14], [15], [16]). Primary DENV infection models were developed by considering the effect of antibody immunity [2], [12], CTL immunity [8], [9], [11], both antibody and CTL immunities [6], [13], [14], [15], both innate and CTL immunities [10] and both innate and antibody immunities [4].

Nikin-Beers and Ciupe [7] introduced a target cell-limited model for primary DENV infection under the effect of CTL immune response as:

$$\text{Uninfected monocytes}\text{: }\;\frac{dU}{dt}=-\underset{\text{Infectious transmission}}{\underset{︸}{\alpha UD}},\text{Infected monocytes}\text{: }\;\frac{dI}{dt}=\underset{\text{Infectious transmission}}{\underset{︸}{\alpha UD}}-\underset{\text{Natural death}}{\underset{︸}{\mu I}}-\underset{\text{Killing of infected cells}}{\underset{︸}{\kappa IC}},\text{Free DENV particles}\text{: }\;\frac{dD}{dt}=\underset{\text{Burst size}}{\underset{︸}{\eta I}}-\underset{\text{Natural death}}{\underset{︸}{\beta D}},\text{CTLs}\text{: }\;\frac{dC}{dt}=\underset{\text{Production of CTLs}}{\underset{︸}{\varkappa }}+\underset{\text{CTLs expansion}}{\underset{︸}{\gamma IC}}-\underset{\text{Natural death}}{\underset{︸}{\nu C}},$$

where C=C(t) is the concentration of CTLs. Parameters *ρ*, *σ*, *α*, *μ*, *η*, *β*, *κ*, *γ* and *ν* are positive.

#### 1.1.2 Secondary DENV infection models

Mathematical models of secondary DENV infection were recently introduced in several works (see e.g., [17], [18], [19], [20], [21], [22]). The effect of immune response was considered in the secondary DENV infection models such as: antibody immune response [17], [18], [19], [21], CTL immune response [7], [13], both antibody and CTL immune responses [20], [22].

In [7], a target cell-limited model for the secondary DENV infection with non-specific and strain-specific CTLs was formulated as:

(1) $$\text{Uninfected monocytes}\text{: }\;\frac{dU}{dt}=-\underset{\text{Infectious transmission}}{\underset{︸}{\alpha UD}},$$

(2) $$\text{Infected monocytes}\text{: }\;\frac{dI}{dt}=\underset{\text{Infectious transmission}}{\underset{︸}{\alpha UD}}-\underset{\text{Natural death}}{\underset{︸}{\mu I}}-\underset{\text{Killing of infected cells by CTLs}}{\underset{︸}{(\kappa_{1}C_{1}+\kappa_{2}C_{2})I}},$$

(3) $$\text{Free DENV particles}\text{: }\;\frac{dD}{dt}=\underset{\text{Burst size}}{\underset{︸}{\eta I}}-\underset{\text{Natural death}}{\underset{︸}{\beta D}},$$

(4) $$\text{Non-specific CTLs}\text{: }\;\frac{dC_{1}}{dt}=\underset{\text{Production of CTLs}}{\underset{︸}{\varkappa }}+\underset{\text{CTLs expansion}}{\underset{︸}{\gamma_{1}IC_{1}}}-\underset{\text{Natural death}}{\underset{︸}{\nu C_{1}}},$$

(5) $$\text{Strain-specific CTLs}\text{: }\;\frac{dC_{2}}{dt}=\underset{\text{Production of CTLs}}{\underset{︸}{\varkappa }}+\underset{\text{CTLs expansion}}{\underset{︸}{\gamma_{2}IC_{2}}}-\underset{\text{Natural death}}{\underset{︸}{\nu C_{2}}}.$$

Here, it was assumed that CTLs specific to primary DENV infection are being produced by immunological memory. In model (1)-(5) we noted the following:

(i) The regeneration and death of the uninfected monocytes are not included. However, in the literature, several DENV infection models considered the regeneration and death of the uninfected monocytes (see e.g., [17], [19], [20], [21]). The population dynamics equation for the uninfected monocytes was given as:

$$\frac{dU}{dt}=\rho -\sigma U-\alpha UD,$$

where *ρ* and *σU* represent the regeneration and death rates of the uninfected monocytes, respectively.

(ii) Both non-specific CTLs and strain-specific CTLs have the same regeneration rate *ϰ*. However, these rates may be differ.

(iii) The death rates of the non-specific CTLs and strain-specific CTLs are equal. However, these rates may be not equal.

The paper aims to formulate two models for secondary DENV infection, incorporating non-specific and strain-specific CTLs. The second model extends the first one by introducing two classes of DENV-infected cells: latently infected cells (containing DENV but not actively producing them) and actively infected cells (producing DENV). The key contributions of our study encompass:

(C1) Considering the regeneration rate and death rate of uninfected monocytes;

(C2) Assuming distinct regeneration rates for non-specific CTLs and strain-specific CTLs;

(C3) Considering different death rates for non-specific CTLs and strain-specific CTLs;

(C4) Investigating the fundamental and global properties of the models;

(C5) Conducting sensitivity analysis;

(C6) Validating theoretical findings through numerical simulations.

## 2 Model with non-specific and strain-specific CTLs

We formulate virus dynamics model with non-specific CTLs ($C_{1})$ and strain-specific CTLs ($C_{2}$):

(6) $$\frac{dU}{dt}=\rho -\sigma U-\alpha UD,$$

(7) $$\frac{dI}{dt}=\alpha UD-\mu I-\kappa_{1}IC_{1}-\kappa_{2}IC_{2},$$

(8) $$\frac{dD}{dt}=\eta I-\beta D,$$

(9) $$\frac{dC_{1}}{dt}=\varkappa_{1}+\gamma_{1}IC_{1}-\nu_{1}C_{1},$$

(10) $$\frac{dC_{2}}{dt}=\varkappa_{2}+\gamma_{2}IC_{2}-\nu_{2}C_{2}.$$

We note that in the absence of DENV infection, there are $\varkappa_{1}/\nu_{1}$ non-specific CTLs and $\varkappa_{2}/\nu_{2}$ strain-specific CTLs available with $\varkappa_{1}$ and $\varkappa_{2}$ being the sources [7]. The initial conditions of system (6)-(10) are

(11) $$U(0)>0,\;I(0)\geq 0,\;D(0)\geq 0,\;C_{1}(0)>0,\;C_{2}(0)>0.$$

We can see that, the right-hand side functions of (6)-(10) satisfy Lipschitz condition, then system (6)-(10) with initial (11) has a unique solution for t≥0.

### 2.1 Preliminary results

First, we determine a compact region for the concentrations of the model's compartments to guarantee that our suggested model is biologically realistic. It is especially important to avoid negative or unbounded concentrations.

Let $\ell_{i}>0$, i=1,2,3,4 be defined as:

$$\ell_{1}=\frac{\rho }{\phi }+\frac{\kappa_{1}\varkappa_{1}}{\gamma_{1}\phi }+\frac{\kappa_{2}\varkappa_{2}}{\gamma_{2}\phi },\;\ell_{2}=\frac{2\eta }{\mu }\ell_{1},\;\ell_{3}=\frac{\gamma_{1}}{\kappa_{1}}\ell_{1}\text{ and }\ell_{4}=\frac{\gamma_{2}}{\kappa_{2}}\ell_{1},$$

where $\phi =\min\{\sigma ,\frac{1}{2}\mu ,\beta ,\nu_{1},\nu_{2}\}$. We define a region O as:

$$O=\left\{(U,I,D,C_{1},C_{2})\in R_{\geq 0}^{5}:0\leq U,I\leq \ell_{1},0\leq D\leq \ell_{2},0\leq C_{1}\leq \ell_{3},0\leq C_{2}\leq \ell_{4}\right\},$$

and $\overset{˚}{O}$ be the interior of O.

Lemma 1 *Solutions of system* (6) *-* (10) *remain positive and bounded within the set* O *.*

**Proof**. Initially,

$$\frac{dU}{dt}{|}_{U=0}=\rho >0,\frac{dI}{dt}{|}_{I=0}=\alpha UD\geq 0\;\text{ for}\;\;U,D\geq 0,\frac{dD}{dt}{|}_{D=0}=\eta I\geq 0\;\text{ for}\;\;I\geq 0,\frac{dC_{1}}{dt}{|}_{C_{1}=0}=\varkappa_{1}>0,\frac{dC_{2}}{dt}{|}_{C_{2}=0}=\varkappa_{2}>0.$$

Thus, all solutions of system (6)-(10) with initial $(U(0),I(0),D(0),C_{1}(0),C_{2}(0))\in R_{\geq 0}^{5}$ satisfy $(U(t),I(t),D(t),C_{1}(t),C_{2}(t))\in R_{\geq 0}^{5}$. Moreover, define a function Ϝ(t) as:

$$Ϝ=U+I+\frac{\mu }{2\eta }D+\frac{\kappa_{1}}{\gamma_{1}}C_{1}+\frac{\kappa_{2}}{\gamma_{2}}C_{2},$$

then

$$\frac{dϜ}{dt}=\rho -\sigma U-\alpha UD+\left(\alpha UD-\mu I-\kappa_{1}IC_{1}-\kappa_{2}IC_{2}\right)+\frac{\mu }{2\eta }\left(\eta I-\beta D\right)+\frac{\kappa_{1}}{\gamma_{1}}\left(\varkappa_{1}+\gamma_{1}IC_{1}-\nu_{1}C_{1}\right)+\frac{\kappa_{2}}{\gamma_{2}}\left(\varkappa_{2}+\gamma_{2}IC_{2}-\nu_{2}C_{2}\right)=\rho +\frac{\kappa_{1}\varkappa_{1}}{\gamma_{1}}+\frac{\kappa_{2}\varkappa_{2}}{\gamma_{2}}-\sigma U-\frac{\mu }{2}I-\frac{\mu \beta }{2\eta }D-\frac{\kappa_{1}{\text{ν}}_{1}}{\gamma_{1}}C_{1}-\frac{\kappa_{2}{\text{ν}}_{2}}{\gamma_{2}}C_{2}\leq \rho +\frac{\kappa_{1}\varkappa_{1}}{\gamma_{1}}+\frac{\kappa_{2}\varkappa_{2}}{\gamma_{2}}-\phi \left(U+I+\frac{\mu }{2\eta }D+\frac{\kappa_{1}}{\gamma_{1}}C_{1}+\frac{\kappa_{2}}{\gamma_{2}}C_{2}\right)=\rho +\frac{\kappa_{1}\varkappa_{1}}{\gamma_{1}}+\frac{\kappa_{2}\varkappa_{2}}{\gamma_{2}}-\phi Ϝ.$$

Hence, $Ϝ(t)\leq \ell_{1}$, if $Ϝ(0)\leq \ell_{1}$. This implies that $0\leq U(t),I(t)\leq \ell_{1}$, $0\leq D(t)\leq \ell_{2}$, $0\leq C_{1}(t)\leq \ell_{3}$ and $0\leq C_{2}(t)\leq \ell_{4}$ if $0\leq U(0)+I(0)+\frac{\mu }{2\eta }D(0)+\frac{\kappa_{1}}{\gamma_{1}}C_{1}(0)+\frac{\kappa_{2}}{\gamma_{2}}C_{2}(0)\leq \ell_{1}$. □

Lemma 2 *A threshold parameter* $R_{0}>0$ *exists such that: (i) when* $R_{0}\leq 1$ *, a unique uninfected equilibrium* ${\mathrm{EQ}}^{0}$ *exists, and (ii) when* $R_{0}>1$ *, an infected equilibrium* ${\mathrm{EQ}}^{⁎}$ *exists besides* ${\mathrm{EQ}}^{0}$ *.*

**Proof**. Any equilibrium point of the model (6)-(10) satisfies the following equations:

(12) 0=ρ−σU−αUD,

(13) $$0=\alpha UD-\mu I-\kappa_{1}IC_{1}-\kappa_{2}IC_{2},$$

(14) 0=ηI−βD,

(15) $$0=\varkappa_{1}+\gamma_{1}IC_{1}-\nu_{1}C_{1},$$

(16) $$0=\varkappa_{2}+\gamma_{2}IC_{2}-\nu_{2}C_{2}.$$

From Eqs. (12), (13), (15) and (16) we have

(17) $$U=\frac{\rho }{\sigma +\alpha D},\;D=\frac{\eta }{\beta }I,C_{1}=\frac{\varkappa_{1}}{{\text{ν}}_{1}-\gamma_{1}I},\;\;C_{2}=\frac{\varkappa_{2}}{{\text{ν}}_{2}-\gamma_{2}I}.$$

Substituting in Eq. (14) we get

(18) $$\left(-\mu +\frac{\eta \alpha \rho }{\beta \sigma +\eta \alpha I}+\frac{\kappa_{1}\varkappa_{1}}{\gamma_{1}I-{\text{ν}}_{1}}+\frac{\kappa_{2}\varkappa_{2}}{\gamma_{2}I-{\text{ν}}_{2}}\right)I=0.$$

Equation (18) presents two scenarios. The first occurs when I=0, yielding the infection-free equilibrium ${\mathrm{EQ}}^{0}(U^{0},0,0,C_{1}^{0},C_{2}^{0})$. Here, $U^{0}=\frac{\rho }{\sigma }$, $C_{1}^{0}=\frac{\varkappa_{1}}{{\text{ν}}_{1}}$ and $C_{2}^{0}=\frac{\varkappa_{2}}{{\text{ν}}_{2}}$.

The second possibility of Eq. (18) arises when I≠0, and it is expressed as:

$$-\mu +\frac{\eta \alpha \rho }{\beta \sigma +\eta \alpha I}+\frac{\kappa_{1}\varkappa_{1}}{\gamma_{1}I-{\text{ν}}_{1}}+\frac{\kappa_{2}\varkappa_{2}}{\gamma_{2}I-{\text{ν}}_{2}}=0,$$

which gives

(19) $$\frac{a_{3}I^{3}+a_{2}I^{2}+a_{1}I+a_{0}}{\left(\beta \sigma +\eta \alpha I)(\gamma_{1}I-{\text{ν}}_{1})(\gamma_{2}I-{\text{ν}}_{2}\right)}=0,$$

where

$$a_{3}=\mu \eta \alpha \gamma_{1}\gamma_{2},a_{2}=-\kappa_{2}\varkappa_{2}\eta \alpha \gamma_{1}-\kappa_{1}\varkappa_{1}\eta \alpha \gamma_{2}+\mu \gamma_{1}\gamma_{2}\beta \sigma -\eta \alpha \gamma_{1}\gamma_{2}\rho -\mu \eta \alpha \gamma_{2}\nu_{1}-\mu \eta \alpha \gamma_{1}\nu_{2},a_{1}=-\kappa_{2}\varkappa_{2}\gamma_{1}\beta \sigma -\kappa_{1}\varkappa_{1}\gamma_{2}\beta \sigma +\kappa_{2}\varkappa_{2}\eta \alpha \nu_{1}-\mu \gamma_{2}\beta \sigma \nu_{1}+\eta \alpha \gamma_{2}\rho \nu_{1}+\kappa_{1}\varkappa_{1}\eta \alpha \nu_{2}-\mu \gamma_{1}\beta \sigma \nu_{2}+\eta \alpha \gamma_{1}\rho \nu_{2}+\mu \eta \alpha \nu_{1}\nu_{2},a_{0}=\kappa_{2}\varkappa_{2}\beta \sigma \nu_{1}+\kappa_{1}\varkappa_{1}\beta \sigma \nu_{2}+\mu \beta \sigma \nu_{1}\nu_{2}-\eta \alpha \rho \nu_{1}\nu_{2}.$$

We define a function $\Phi (I)=a_{3}I^{3}+a_{2}I^{2}+a_{1}I+a_{0}$, then we get

$$\Phi (0)=-\beta \sigma (\kappa_{2}\varkappa_{2}\nu_{1}+\kappa_{1}\varkappa_{1}\nu_{2}+\mu \nu_{1}\nu_{2})\left(\frac{\eta \alpha \rho }{\mu \sigma \beta \left(\frac{\kappa_{1}\varkappa_{1}}{{\text{ν}}_{1}\mu }+\frac{\kappa_{2}\varkappa_{2}}{{\text{ν}}_{2}\mu }+1\right)}-1\right),\Phi \left(\frac{{\text{ν}}_{1}}{\gamma_{1}}\right)=\frac{\kappa_{1}\varkappa_{1}\gamma_{2}(\beta \sigma \gamma_{1}+\alpha {\text{ν}}_{1}\eta )}{\gamma_{1}}\left(\frac{{\text{ν}}_{2}}{\gamma_{2}}-\frac{{\text{ν}}_{1}}{\gamma_{1}}\right),\Phi \left(\frac{{\text{ν}}_{2}}{\gamma_{2}}\right)=\frac{\kappa_{2}\varkappa_{2}\gamma_{1}(\beta \sigma \gamma_{2}+\alpha {\text{ν}}_{2}\eta )}{\gamma_{2}}\left(\frac{{\text{ν}}_{1}}{\gamma_{1}}-\frac{{\text{ν}}_{2}}{\gamma_{2}}\right),\lim_{I\to \infty }\Phi (I)=\infty .$$

If the condition

(C) $$\frac{\eta \alpha \rho }{\mu \sigma \beta \left(\frac{\kappa_{1}\varkappa_{1}}{{\text{ν}}_{1}\mu }+\frac{\kappa_{2}\varkappa_{2}}{{\text{ν}}_{2}\mu }+1\right)}>1$$

is met, Φ(0) will be less than 0. Observe that:

$$\frac{{\text{ν}}_{2}}{\gamma_{2}}>\frac{{\text{ν}}_{1}}{\gamma_{1}}⟹\Phi \left(\frac{{\text{ν}}_{1}}{\gamma_{1}}\right)>0\text{ and }\Phi \left(\frac{{\text{ν}}_{2}}{\gamma_{2}}\right)<0,\frac{{\text{ν}}_{2}}{\gamma_{2}}<\frac{{\text{ν}}_{1}}{\gamma_{1}}⟹\Phi \left(\frac{{\text{ν}}_{1}}{\gamma_{1}}\right)<0\text{ and }\Phi \left(\frac{{\text{ν}}_{2}}{\gamma_{2}}\right)>0.$$

Hence,

$$\Phi \left(\min\left\{\frac{{\text{ν}}_{1}}{\gamma_{1}},\frac{{\text{ν}}_{2}}{\gamma_{2}}\right\}\right)>0\text{ and }\Phi \left(\max\left\{\frac{{\text{ν}}_{1}}{\gamma_{1}},\frac{{\text{ν}}_{2}}{\gamma_{2}}\right\}\right)<0.$$

If condition (C) holds, then Φ(0)<0 and Eq. (19) has three positive roots

$$I^{⁎}\in \left(0,\min\left\{\frac{{\text{ν}}_{1}}{\gamma_{1}},\frac{{\text{ν}}_{2}}{\gamma_{2}}\right\}\right),\overset{¯}{I}\in \left(\min\left\{\frac{{\text{ν}}_{1}}{\gamma_{1}},\frac{{\text{ν}}_{2}}{\gamma_{2}}\right\},\max\left\{\frac{{\text{ν}}_{1}}{\gamma_{1}},\frac{{\text{ν}}_{2}}{\gamma_{2}}\right\}\right),\overset{˜}{I}\in \left(\max\left\{\frac{{\text{ν}}_{1}}{\gamma_{1}},\frac{{\text{ν}}_{2}}{\gamma_{2}}\right\},\infty \right).$$

We observe from Eq. (17) that the solution $\overset{¯}{I}$ results $C_{1}<0$ or $C_{2}<0$, moreover, $\overset{˜}{I}$ yields $C_{1}<0$ and $C_{2}<0$. Hence, the sole admissible solution is $I^{⁎}$, which results in

$$U^{⁎}=\frac{\rho }{\sigma +\alpha D^{⁎}}>0,\;D^{⁎}=\frac{\eta }{\beta }I^{⁎}>0,C_{1}^{⁎}=\frac{\varkappa_{1}}{{\text{ν}}_{1}-\gamma_{1}I^{⁎}}>0,\;\;C_{2}^{⁎}=\frac{\varkappa_{2}}{{\text{ν}}_{2}-\gamma_{2}I^{⁎}}>0.$$

Accordingly, the basic reproduction number $R_{0}$ can be defined as follows:

$$R_{0}=\frac{\eta \alpha U_{0}}{\mu \beta \left(\frac{\kappa_{1}C_{1}^{0}}{\mu }+\frac{\kappa_{2}C_{2}^{0}}{\mu }+1\right)}.$$

Then, the infected equilibrium ${\mathrm{EQ}}^{⁎}(U^{⁎},I^{⁎},D^{⁎},C_{1}^{⁎},C_{2}^{⁎})$ is present when $R_{0}>1$. It is worth noting that the next-generation matrix approach [23] or the local stability of the uninfected equilibrium ${\mathrm{EQ}}^{0}$ can also be employed to determine $R_{0}$. This basic reproduction number is defined as the quantity of infected cells produced by one infected cell in its lifespan in a fully susceptible environment. □

### 2.2 Global stability

The global stability analysis is fundamental for comprehending and predicting the behavior of systems described by mathematical models. In this section we investigate the global stability of the two equilibria of system (6)-(10). The proof is derived from the method of constructing Lyapunov functions for viral infection models (see e.g., [24], [25], [26]). Define a function $\Theta_{j}(U,I,D,C_{1},C_{2})$ and let ${\overset{˜}{\Gamma }}_{j}$ be the largest invariant subset of $\Gamma_{j}=\left\{(U,I,D,C_{1},C_{2}):\frac{d\Theta_{j}}{dt}=0\right\}$, j=0,1. During this section, we utilize the arithmetic-mean-geometric-mean inequality

(20) $$\frac{1}{n}\sum_{i=1}^{n}k_{i}\geq \sqrt[{n}]{\prod_{i=1}^{n}k_{i}},\;k_{i}\geq 0,\;i=1,2,...,n.$$

The following finding indicates that, independent of the beginning circumstances (any illness phases), the DENV infection is anticipated to die out when $R_{0}\leq 1$.

Theorem 1 *(i) In the case that* $R_{0}\leq 1$ *, the uninfected equilibrium* ${\mathrm{EQ}}^{0}(U^{0},0,0,C_{1}^{0},C_{2}^{0})$ *is global asymptotic stability (G.A.S) within the set* O *, and (ii) if* $R_{0}>1$ *,* ${\mathrm{EQ}}^{0}$ *becomes unstable.*

**Proof.** (i) Define

$$\Theta_{0}=U^{0}\left(\frac{U}{U^{0}}-1-\ln\left(\frac{U}{U^{0}}\right)\right)+I+\frac{\alpha U^{0}}{\beta }D+\frac{\kappa_{1}}{\gamma_{1}}C_{1}^{0}\left(\frac{C_{1}}{C_{1}^{0}}-1-\ln\left(\frac{C_{1}}{C_{1}^{0}}\right)\right)+\frac{\kappa_{2}}{\gamma_{2}}C_{2}^{0}\left(\frac{C_{2}}{C_{2}^{0}}-1-\ln\left(\frac{C_{2}}{C_{2}^{0}}\right)\right).$$

We observe that $\Theta_{0}(U,I,D,C_{1},C_{2})>0$ for all $(U,I,D,C_{1},C_{2})>0$ and $\Theta_{0}(U^{0},0,0,C_{1}^{0},C_{2}^{0})=0$. Calculating $\frac{d\Theta_{0}}{dt}$ along the solutions of (6)-(10) as:

$$\frac{d\Theta_{0}}{dt}=\left(1-\frac{U^{0}}{U}\right)\left(\rho -\sigma U-\alpha UD\right)+\alpha UD-\mu I-\kappa_{1}IC_{1}-\kappa_{2}IC_{2}+\frac{\alpha U^{0}}{\beta }\left(\eta I-\beta D\right)+\frac{\kappa_{1}}{\gamma_{1}}\left(1-\frac{C_{1}^{0}}{C_{1}}\right)(\varkappa_{1}+\gamma_{1}IC_{1}-\nu_{1}C_{1})+\frac{\kappa_{2}}{\gamma_{2}}\left(1-\frac{C_{2}^{0}}{C_{2}}\right)(\varkappa_{2}+\gamma_{2}IC_{2}-\nu_{2}C_{2})=\left(1-\frac{U^{0}}{U}\right)\left(\rho -\sigma U\right)-\mu I+\frac{\alpha U^{0}}{\beta }\eta I+\frac{\kappa_{1}}{\gamma_{1}}\left(1-\frac{C_{1}^{0}}{C_{1}}\right)(\varkappa_{1}-\nu_{1}C_{1})-\kappa_{1}C_{1}^{0}I+\frac{\kappa_{2}}{\gamma_{2}}\left(1-\frac{C_{2}^{0}}{C_{2}}\right)(\varkappa_{2}-\nu_{2}C_{2})-\kappa_{2}C_{2}^{0}I.$$

Using $\rho =\sigma U^{0}$, $\varkappa_{1}=\nu_{1}C_{1}^{0}$ and $\varkappa_{2}=\nu_{2}C_{2}^{0}$ we obtain

$$\frac{d\Theta_{0}}{dt}=-{\frac{\sigma \left(U-U^{0}\right)}{U}}^{2}-\frac{\kappa_{1}{\text{ν}}_{1}}{\gamma_{1}}\frac{{\left(C_{1}-C_{1}^{0}\right)}^{2}}{C_{1}}-\frac{\kappa_{2}{\text{ν}}_{2}}{\gamma_{2}}\frac{{\left(C_{2}-C_{2}^{0}\right)}^{2}}{C_{2}}+\left(\frac{\alpha U^{0}}{\beta }\eta -\mu -\kappa_{1}C_{1}^{0}-\kappa_{2}C_{2}^{0}\right)I=-{\frac{\sigma \left(U-U^{0}\right)}{U}}^{2}-\frac{\kappa_{1}{\text{ν}}_{1}}{\gamma_{1}}\frac{{\left(C_{1}-C_{1}^{0}\right)}^{2}}{C_{1}}-\frac{\kappa_{2}{\text{ν}}_{2}}{\gamma_{2}}\frac{{\left(C_{2}-C_{2}^{0}\right)}^{2}}{C_{2}}+\frac{\eta \alpha U_{0}}{\beta R_{0}}\left(R_{0}-1\right)I.$$

If $R_{0}\leq 1$, then $\frac{d\Theta_{0}}{dt}\leq 0$ holds for all $U,I,D,C_{1},C_{2}\in (0,\infty )$. Furthermore, $\frac{d\Theta_{0}}{dt}=0$ when $U(t)=U^{0}$, $C_{1}(t)=C_{1}^{0}$, $C_{2}(t)=C_{2}^{0}$ and I(t)=0 for all *t*. The solutions to the system described in equations (6)-(10) exhibit convergence towards ${\overset{˜}{\Gamma }}_{0}$ [27]. The set ${\overset{˜}{\Gamma }}_{0}$ encompasses elements that satisfy $U(t)=U^{0}$, $C_{1}(t)=C_{1}^{0}$, $C_{2}(t)=C_{2}^{0}$ and I(t)=0. From Eq. (7), we deduce that

$$0=\frac{dI}{dt}=\alpha U_{0}D⟹D(t)=0.$$

Therefore, ${\overset{˜}{\Gamma }}_{0}$ is a singleton $\left\{{\mathrm{EQ}}^{0}\right\}$. By applying the LaSalle-Lyapunov (L-LAS) theorem [28], [29], [30], it can be established that ${\mathrm{EQ}}^{0}$ is G.A.S in O.

(ii) The Jacobian matrix $J_{1}=J_{1}(U,I,D,C_{1},C_{2})$ of system (6)-(10) is calculated as:

$$J_{1}=\left(\begin{array}{ccccc} -\sigma -D\alpha \; & 0\; & -\alpha U\; & 0\; & 0\\ \alpha D\; & -\mu -\kappa_{1}C_{1}-\kappa_{2}C_{2}\; & \alpha U\; & -\kappa_{1}I\; & -\kappa_{2}I\\ 0\; & \eta \; & -\beta \; & 0\; & 0\\ 0\; & \gamma_{1}C_{1}\; & 0\; & \gamma_{1}I-{\text{ν}}_{1}\; & 0\\ 0\; & \gamma_{2}C_{2}\; & 0\; & 0\; & \gamma_{2}I-{\text{ν}}_{2} \end{array}\right),$$

and the characteristic equation at the equilibrium ${\mathrm{EQ}}^{0}$ is expressed as

(21) $$\det(J_{1}-\xi I)=(\xi +\sigma )(\xi +\nu_{1})(\xi +\nu_{2})\left(p_{2}\xi^{2}+p_{1}\xi +p_{0}\right)=0,$$

where *ξ* is the eigenvalue and

$$p_{2}=\sigma \nu_{1}\nu_{2},p_{1}=\sigma \kappa_{2}\varkappa_{2}\nu_{1}+\sigma \kappa_{1}\varkappa_{1}\nu_{2}+\mu \sigma \nu_{1}\nu_{2}+\beta \sigma \nu_{1}\nu_{2},p_{0}=\beta \sigma \kappa_{2}\varkappa_{2}\nu_{1}+\beta \sigma \kappa_{1}\varkappa_{1}\nu_{2}+\mu \beta \sigma \nu_{1}\nu_{2}-\eta \alpha \rho \nu_{1}\nu_{2}=\beta \sigma (\kappa_{2}\varkappa_{2}\nu_{1}+\kappa_{1}\varkappa_{1}\nu_{2}+\mu \nu_{1}\nu_{2})(1-R_{0}).$$

Clearly if $R_{0}>1$, then $p_{0}<0$ and Eq. (21) has a positive root and hence ${\mathrm{EQ}}^{0}$ is unstable. □

According to the results shown below, regardless of the starting circumstances, the DENV infection always occurs when $R_{0}>1$.

Theorem 2 *The infected equilibrium* ${\mathrm{EQ}}^{⁎}(U^{⁎},I^{⁎},D^{⁎},C_{1}^{⁎},C_{2}^{⁎})$ *is G.A.S in* $\overset{˚}{O}$ *if* $R_{0}>1$ *.*

**Proof.** Define

$$\Theta_{1}=U^{⁎}\left(\frac{U}{U^{⁎}}-1-\ln\left(\frac{U}{U^{⁎}}\right)\right)+I^{⁎}\left(\frac{I}{I^{⁎}}-1-\ln\left(\frac{I}{I^{⁎}}\right)\right)+\frac{\alpha U^{⁎}}{\beta }D^{⁎}\left(\frac{D}{D^{⁎}}-1-\ln\left(\frac{D}{D^{⁎}}\right)\right)+\frac{\kappa_{1}}{\gamma_{1}}C_{1}^{⁎}\left(\frac{C_{1}}{C_{1}^{⁎}}-1-\ln\left(\frac{C_{1}}{C_{1}^{⁎}}\right)\right)+\frac{\kappa_{2}}{\gamma_{2}}C_{2}^{⁎}\left(\frac{C_{2}}{C_{2}^{⁎}}-1-\ln\left(\frac{C_{2}}{C_{2}^{⁎}}\right)\right).$$

Clearly, $\Theta_{1}(U,I,D,C_{1},C_{2})>0$ for all $(U,I,D,C_{1},C_{2})>0$ and $\Theta_{1}(U^{⁎},I^{⁎},D^{⁎},C_{1}^{⁎},C_{2}^{⁎})=0$. Calculating $\frac{d\Theta_{1}}{dt}$ along the trajectories of (6)-(10):

$$\frac{d\Theta_{1}}{dt}=\left(1-\frac{U^{⁎}}{U}\right)\left(\rho -\sigma U-\alpha UD\right)+\left(1-\frac{I^{⁎}}{I}\right)\left(\alpha UD-\mu I-\kappa_{1}IC_{1}-\kappa_{2}IC_{2}\right)+\frac{\alpha U^{⁎}}{\beta }\left(1-\frac{D^{⁎}}{D}\right)\left(\eta I-\beta D\right)+\frac{\kappa_{1}}{\gamma_{1}}\left(1-\frac{C_{1}^{⁎}}{C_{1}}\right)(\varkappa_{1}+\gamma_{1}IC_{1}-\nu_{1}C_{1})+\frac{\kappa_{2}}{\gamma_{2}}\left(1-\frac{C_{2}^{⁎}}{C_{2}}\right)(\varkappa_{2}+\gamma_{2}IC_{2}-\nu_{2}C_{2})=\left(1-\frac{U^{⁎}}{U}\right)(\rho -\sigma U)-\mu I-\frac{\alpha UDI^{⁎}}{I}+\mu I^{⁎}+\kappa_{1}I^{⁎}C_{1}+\kappa_{2}I^{⁎}C_{2}+\frac{\alpha U^{⁎}}{\beta }\eta I-\frac{\alpha U^{⁎}\eta }{\beta }\frac{D^{⁎}I}{D}+\alpha U^{⁎}D^{⁎}+\frac{\kappa_{1}}{\gamma_{1}}\left(1-\frac{C_{1}^{⁎}}{C_{1}}\right)(\varkappa_{1}-\nu_{1}C_{1})-\kappa_{1}C_{1}^{⁎}I+\frac{\kappa_{2}}{\gamma_{2}}\left(1-\frac{C_{2}^{⁎}}{C_{2}}\right)(\varkappa_{2}-\nu_{2}C_{2})-\kappa_{2}C_{2}^{⁎}I.$$

Applying the equilibrium conditions

$$\rho =\sigma U^{⁎}+\alpha U^{⁎}D^{⁎},\;\;\mu I^{⁎}=\alpha U^{⁎}D^{⁎}-\kappa_{1}I^{⁎}C_{1}^{⁎}-\kappa_{2}I^{⁎}C_{2}^{⁎},\;\;\eta I^{⁎}=\beta D^{⁎},\varkappa_{1}=\nu_{1}C_{1}^{⁎}-\gamma_{1}I^{⁎}C_{1}^{⁎},\varkappa_{2}=\nu_{2}C_{2}^{⁎}-\gamma_{2}I^{⁎}C_{2}^{⁎},$$

we get

$$-\mu I+\frac{\alpha U^{⁎}}{\beta }\eta I-\kappa_{1}C_{1}^{⁎}I-\kappa_{2}C_{2}^{⁎}I=\left(-\mu I^{⁎}+\frac{\alpha U^{⁎}}{\beta }\eta I^{⁎}-\kappa_{1}C_{1}^{⁎}I^{⁎}-\kappa_{2}C_{2}^{⁎}I^{⁎}\right)\frac{I}{I^{⁎}}=\left(-\mu I^{⁎}+\alpha U^{⁎}D^{⁎}-\kappa_{1}C_{1}^{⁎}I^{⁎}-\kappa_{2}C_{2}^{⁎}I^{⁎}\right)\frac{I}{I^{⁎}}=0,$$

and

$$\frac{d\Theta_{1}}{dt}=\left(1-\frac{U^{⁎}}{U}\right)(\sigma U^{⁎}+\alpha U^{⁎}D^{⁎}-\sigma U)-\alpha U^{⁎}D^{⁎}\frac{UDI^{⁎}}{U^{⁎}D^{⁎}I}+\alpha U^{⁎}D^{⁎}-\kappa_{1}I^{⁎}C_{1}^{⁎}-\kappa_{2}I^{⁎}C_{2}^{⁎}+\kappa_{1}I^{⁎}C_{1}+\kappa_{2}I^{⁎}C_{2}-\alpha U^{⁎}D^{⁎}\frac{D^{⁎}I}{DI^{⁎}}+\alpha U^{⁎}D^{⁎}+\frac{\kappa_{1}}{\gamma_{1}}\left(1-\frac{C_{1}^{⁎}}{C_{1}}\right)(\nu_{1}C_{1}^{⁎}-\gamma_{1}I^{⁎}C_{1}^{⁎}-\nu_{1}C_{1})+\frac{\kappa_{2}}{\gamma_{2}}\left(1-\frac{C_{2}^{⁎}}{C_{2}}\right)(\nu_{2}C_{2}^{⁎}-\gamma_{2}I^{⁎}C_{2}^{⁎}-\nu_{2}C_{2}).$$

Then

$$\frac{d\Theta_{1}}{dt}=-\frac{\sigma {\left(U-U^{⁎}\right)}^{2}}{U}+\alpha U^{⁎}D^{⁎}\left(3-\frac{U^{⁎}}{U}-\frac{UDI^{⁎}}{U^{⁎}D^{⁎}I}-\frac{D^{⁎}I}{DI^{⁎}}\right)-\frac{{\text{ν}}_{1}\kappa_{1}}{\gamma_{1}}\frac{{\left(C_{1}-C_{1}^{⁎}\right)}^{2}}{C_{1}}-\frac{{\text{ν}}_{2}\kappa_{2}}{\gamma_{2}}\frac{{\left(C_{2}-C_{2}^{⁎}\right)}^{2}}{C_{2}}+\kappa_{1}I^{⁎}C_{1}^{⁎}\left(-2+\frac{C_{1}^{⁎}}{C_{1}}+\frac{C_{1}}{C_{1}^{⁎}}\right)+\kappa_{2}I^{⁎}C_{2}^{⁎}\left(-2+\frac{C_{2}^{⁎}}{C_{2}}+\frac{C_{2}}{C_{2}^{⁎}}\right)=-\frac{\sigma {\left(U-U^{⁎}\right)}^{2}}{U}+\alpha U^{⁎}D^{⁎}\left(3-\frac{U^{⁎}}{U}-\frac{UDI^{⁎}}{U^{⁎}D^{⁎}I}-\frac{D^{⁎}I}{DI^{⁎}}\right)-\frac{{\text{ν}}_{1}\kappa_{1}}{\gamma_{1}}\frac{{\left(C_{1}-C_{1}^{⁎}\right)}^{2}}{C_{1}}-\frac{{\text{ν}}_{2}\kappa_{2}}{\gamma_{2}}\frac{{\left(C_{2}-C_{2}^{⁎}\right)}^{2}}{C_{2}}+\kappa_{1}I^{⁎}\frac{{\left(C_{1}-C_{1}^{⁎}\right)}^{2}}{C_{1}}+\kappa_{2}I^{⁎}\frac{{\left(C_{2}-C_{2}^{⁎}\right)}^{2}}{C_{2}}.$$

From the equilibrium conditions we have $I^{⁎}-\frac{{\text{ν}}_{1}}{\gamma_{1}}=-\frac{\varkappa_{1}}{\gamma_{1}C_{1}^{⁎}}$ and $I^{⁎}-\frac{{\text{ν}}_{2}}{\gamma_{2}}=-\frac{\varkappa_{2}}{\gamma_{2}C_{2}^{⁎}}$. It follows that

$$\frac{d\Theta_{1}}{dt}=-\frac{\sigma {\left(U-U^{⁎}\right)}^{2}}{U}-\frac{\varkappa_{1}\kappa_{1}}{\gamma_{1}}\frac{{\left(C_{1}-C_{1}^{⁎}\right)}^{2}}{C_{1}C_{1}^{⁎}}-\frac{\varkappa_{2}\kappa_{2}}{\gamma_{2}}\frac{{\left(C_{2}-C_{2}^{⁎}\right)}^{2}}{C_{2}^{⁎}C_{2}}+\alpha U^{⁎}D^{⁎}\left(3-\frac{U^{⁎}}{U}-\frac{UDI^{⁎}}{U^{⁎}D^{⁎}I}-\frac{D^{⁎}I}{DI^{⁎}}\right).$$

Applying inequality (20) we get

$$\frac{1}{3}\left(\frac{U^{⁎}}{U}+\frac{UDI^{⁎}}{U^{⁎}D^{⁎}I}+\frac{D^{⁎}I}{DI^{⁎}}\right)\geq 1.$$

Consequently, $\frac{d\Theta_{1}}{dt}\leq 0$ for all $U,I,D,C_{1},C_{2}>0$. Moreover, $\frac{d\Theta_{1}}{dt}=0$ when $\left(U,I,D,C_{1},C_{2})=(U^{⁎},I^{⁎},D^{⁎},C_{1}^{⁎},C_{2}^{⁎}\right)$ and thus ${\overset{˜}{\Gamma }}_{1}=\{{\mathrm{EQ}}^{⁎}\}$. L-LAS theorem implies that ${\mathrm{EQ}}^{⁎}$ is G.A.S in $\overset{˚}{O}$. □

## 3 Model with latency

Throughout this section, we consider that cells infected with the DENV virus can exist in two states, namely latent or active. We posit that a proportion θ∈(0,1) of DENV-infected cells transition to an active state, while the remaining fraction (1−θ) becomes latent. Let L=L(t) represent the concentration of latently DENV-infected cells at time *t*. The model incorporating latency can be expressed as:

(22) $$\frac{dU}{dt}=\rho -\sigma U-\alpha UD,$$

(23) $$\frac{dL}{dt}=(1-\theta )\alpha UD-(\delta +\lambda )L,$$

(24) $$\frac{dI}{dt}=\theta \alpha UD+\delta L-\mu I-\kappa_{1}IC_{1}-\kappa_{2}IC_{2},$$

(25) $$\frac{dD}{dt}=\eta I-\beta D,$$

(26) $$\frac{dC_{1}}{dt}=\varkappa_{1}+\gamma_{1}IC_{1}-\nu_{1}C_{1},$$

(27) $$\frac{dC_{2}}{dt}=\varkappa_{2}+\gamma_{2}IC_{2}-\nu_{2}C_{2}.$$

Latent DENV-infected cells activate at a rate of *δL* and experience mortality at a rate of *λL*.

The initial conditions of system (22)-(27) are

(28) $$U(0)>0,\;L(0)\geq 0,\;I(0)\geq 0,\;D(0)\geq 0,\;C_{1}(0)>0,\;C_{2}(0)>0.$$

We can see that, the right-hand side functions of (22)-(27) satisfy Lipschitz condition, then system (22)-(27) with initial (28) has a unique solution for t≥0.

### 3.1 Preliminary results

Define positive constants $\ell_{i}^{L}$, where i=1,2,3,4, as follows:

$$\ell_{1}^{L}=\frac{\rho }{\phi^{L}}+\frac{\kappa_{1}\varkappa_{1}}{\gamma_{1}\phi^{L}}+\frac{\kappa_{2}\varkappa_{2}}{\gamma_{2}\phi^{L}}\text{, }\ell_{2}^{L}=\frac{2\eta }{\mu }\ell_{1}^{L}\text{, }\ell_{3}^{L}=\frac{\gamma_{1}}{\kappa_{1}}\ell_{1}^{L}\text{ and }\ell_{4}^{L}=\frac{\gamma_{2}}{\kappa_{2}}\ell_{1}^{L},$$

where $\phi^{L}=\min\{\sigma ,\lambda ,\frac{1}{2}\mu ,\beta ,\nu_{1},\nu_{2}\}$. Define a domain $O^{L}$ as:

$$O^{L}=\left\{(U,L,I,D,C_{1},C_{2})\in R_{\geq 0}^{6}:0\leq U,L,I\leq \ell_{1}^{L},0\leq D\leq \ell_{2}^{L},0\leq C_{1}\leq \ell_{3}^{L},0\leq C_{2}\leq \ell_{4}^{L}\right\}.$$

Lemma 3 *The solutions to system* (22) *-* (27) *remain positively invariant and bounded within the set* $O^{L}$ *.*

**Proof**. Since

$$\frac{dU}{dt}{|}_{U=0}=\rho >0,\;\;\frac{dL}{dt}{|}_{L=0}=(1-\theta )\alpha UD\geq 0,\;\text{for}\;\;U,D\geq 0,\frac{dI}{dt}{|}_{I=0}=\theta \alpha UD+\delta L\geq 0,\;\text{for}\;\;U,D,L\geq 0,\frac{dD}{dt}{|}_{D=0}=\eta I\geq 0,\;\text{for}\;\;I\geq 0,\frac{dC_{1}}{dt}{|}_{C_{1}=0}=\varkappa_{1}>0,\;\frac{dC_{2}}{dt}{|}_{C_{2}=0}=\varkappa_{2}>0.$$

therefore, all solutions of system (22)-(27) with initial conditions $(U(0),L(0),I(0),D(0),C_{1}(0),C_{2}(0))\in R_{\geq 0}^{6}$ satisfy $(U(t),L(t),I(t),D(t),C_{1}(t),C_{2}(t))\in R_{\geq 0}^{6}$. Let

$${Ϝ}^{L}=U+L+I+\frac{\mu }{2\eta }D+\frac{\kappa_{1}}{\gamma_{1}}C_{1}+\frac{\kappa_{2}}{\gamma_{2}}C_{2},$$

then

$$\frac{d{Ϝ}^{L}}{dt}=\rho -\sigma U-\alpha UD+(1-\theta )\alpha UD-(\delta +\lambda )L+\theta \alpha UD+\delta L-\mu I-\kappa_{1}IC_{1}-\kappa_{2}IC_{2}+\frac{\mu }{2\eta }\left(\eta I-\beta D\right)+\frac{\kappa_{1}}{\gamma_{1}}\left(\varkappa_{1}+\gamma_{1}IC_{1}-\nu_{1}C_{1}\right)+\frac{\kappa_{2}}{\gamma_{2}}\left(\varkappa_{2}+\gamma_{2}IC_{2}-\nu_{2}C_{2}\right)=\rho +\frac{\kappa_{1}\varkappa_{1}}{\gamma_{1}}+\frac{\kappa_{2}\varkappa_{2}}{\gamma_{2}}-\sigma U-\lambda L-\frac{\mu }{2}I-\frac{\mu \beta }{2\eta }D-\frac{\kappa_{1}{\text{ν}}_{1}}{\gamma_{1}}C_{1}-\frac{\kappa_{2}{\text{ν}}_{2}}{\gamma_{2}}C_{2}\leq \rho +\frac{\kappa_{1}\varkappa_{1}}{\gamma_{1}}+\frac{\kappa_{2}\varkappa_{2}}{\gamma_{2}}-\phi^{L}\left(U+L+I+\frac{\mu }{2\eta }D+\frac{\kappa_{1}}{\gamma_{1}}C_{1}+\frac{\kappa_{2}}{\gamma_{2}}C_{2}\right)=\rho +\frac{\kappa_{1}\varkappa_{1}}{\gamma_{1}}+\frac{\kappa_{2}\varkappa_{2}}{\gamma_{2}}-\phi^{L}{Ϝ}^{L}.$$

Hence, ${Ϝ}^{L}(t)\leq \ell_{1}^{L}$, if ${Ϝ}^{L}(0)\leq \ell_{1}^{L}$. This implies that $0\leq U(t),L(t),I(t)\leq \ell_{1}^{L}$, $0\leq D(t)\leq \ell_{2}^{L}$, $0\leq C_{1}(t)\leq \ell_{3}^{L}$ and $0\leq C_{2}(t)\leq \ell_{4}^{L}$ if $0\leq U(0)+L(0)+I(0)+\frac{\mu }{2\eta }D(0)+\frac{\kappa_{1}}{\gamma_{1}}C_{1}(0)+\frac{\kappa_{2}}{\gamma_{2}}C_{2}(0)\leq \ell_{1}^{L}$. □

Lemma 4 *A threshold parameter* $R_{0}^{L}>0$ *exists such that: (i) when* $R_{0}^{L}\leq 1$ *, a unique uninfected equilibrium* ${\mathrm{EQ}}^{0}$ *exists, and (ii) when* $R_{0}^{L}>1$ *, an infected equilibrium* ${\mathrm{EQ}}^{⁎}$ *exists besides* ${\mathrm{EQ}}^{0}$ *.*

**Proof**. The equilibrium points of system (22)-(27) are determined by solving the following equations:

(29) 0=ρ−σU−αUD,

(30) 0=(1−θ)αUD−(δ+λ)L,

(31) $$0=\theta \alpha UD+\delta L-\mu I-\kappa_{1}IC_{1}-\kappa_{2}IC_{2},$$

(32) 0=ηI−βD,

(33) $$0=\varkappa_{1}+\gamma_{1}IC_{1}-\nu_{1}C_{1},$$

(34) $$0=\varkappa_{2}+\gamma_{2}IC_{2}-\nu_{2}C_{2}.$$

From Eqs. (29)-(31) and (33)-(34) we have

(35) $$U=\frac{\rho }{\sigma +\alpha D},\;L=\frac{(1-\theta )\alpha \rho D}{\left(\delta +\lambda )(\sigma +\alpha D\right)},\;D=\frac{\eta I}{\beta },C_{1}=\frac{\varkappa_{1}}{{\text{ν}}_{1}-\gamma_{1}I},\;\;C_{2}=\frac{\varkappa_{2}}{{\text{ν}}_{2}-\gamma_{2}I}.$$

Substituting in Eq. (32) we get

(36) $$\left(-\mu +\frac{\kappa_{1}\varkappa_{1}}{\gamma_{1}I-{\text{ν}}_{1}}+\frac{\kappa_{2}\varkappa_{2}}{\gamma_{2}I-{\text{ν}}_{2}}+\frac{\eta \alpha \rho (\delta +\theta \lambda )}{\left(\sigma \beta +\alpha \eta I)(\delta +\lambda \right)}\right)I=0.$$

Equation (36) presents two scenarios. The rst occurs when I=0, yielding the infection-free equilibrium ${\mathrm{EQ}}^{0}(U^{0},0,0,0,C_{1}^{0},C_{2}^{0})$. Here, $U^{0}=\frac{\rho }{\sigma }$, $C_{1}^{0}=\frac{\varkappa_{1}}{{\text{ν}}_{1}}$ and $C_{2}^{0}=\frac{\varkappa_{2}}{{\text{ν}}_{2}}$. The second possibility of Eq. (36) arises when I≠0, and it is expressed as:

$$-\mu +\frac{\kappa_{1}\varkappa_{1}}{\gamma_{1}I-{\text{ν}}_{1}}+\frac{\kappa_{2}\varkappa_{2}}{\gamma_{2}I-{\text{ν}}_{2}}+\frac{\eta \alpha \rho (\delta +\theta \lambda )}{\left(\sigma \beta +\alpha \eta I)(\delta +\lambda \right)}=0,$$

(37) $$⟹\frac{a_{3}^{L}I^{3}+a_{2}^{L}I^{2}+a_{1}^{L}I+a_{0}^{L}}{\left(\delta +\lambda )(\sigma \beta +\alpha \eta I)(\gamma_{1}I-{\text{ν}}_{1})(\gamma_{2}I-{\text{ν}}_{2}\right)}=0,$$

where

$$a_{3}^{L}=\mu \eta \alpha \gamma_{1}\gamma_{2}(\delta +\lambda ),a_{2}^{L}=-\kappa_{2}\varkappa_{2}\eta \alpha \gamma_{1}(\delta +\lambda )-\gamma_{2}(\kappa_{1}\varkappa_{1}\eta \alpha (\delta +\lambda )+\eta \alpha \gamma_{1}\rho (\delta +\theta \lambda )-\mu (\delta +\lambda )(\gamma_{1}\beta \sigma -\eta \alpha \nu_{1}))\;-\mu \eta \alpha \gamma_{1}\nu_{2}(\delta +\lambda ),a_{1}^{L}=-\kappa_{1}\varkappa_{1}\gamma_{2}\beta \sigma (\delta +\lambda )+\gamma_{2}(-\mu \beta \sigma (\delta +\lambda )+\eta \alpha \rho (\delta +\theta \lambda ))\nu_{1}-\kappa_{2}\varkappa_{2}(\delta +\lambda )(\gamma_{1}\beta \sigma -\eta \alpha \nu_{1})\;+(\kappa_{1}\varkappa_{1}\eta \alpha (\delta +\lambda )+\eta \alpha \gamma_{1}\rho (\delta +\theta \lambda )-\mu (\delta +\lambda )(\gamma_{1}\beta \sigma -\eta \alpha \nu_{1}))\nu_{2},a_{0}^{L}=\kappa_{2}\varkappa_{2}\beta \sigma (\delta +\lambda )\nu_{1}+(\kappa_{1}\varkappa_{1}\beta \sigma (\delta +\lambda )+(\mu \beta \sigma (\delta +\lambda )-\eta \alpha \rho (\delta +\theta \lambda ))\nu_{1})\nu_{2}.$$

Let $\Phi^{L}(I)=a_{3}^{L}I^{3}+a_{2}^{L}I^{2}+a_{1}^{L}I+a_{0}^{L}$, then we get

$$\Phi^{L}(0)=-\beta \sigma (\delta +\lambda )(\kappa_{2}\varkappa_{2}\nu_{1}+\kappa_{1}\varkappa_{1}\nu_{2}+\mu \nu_{1}\nu_{2})\left(\frac{\eta \alpha \rho (\delta +\theta \lambda )}{\mu \sigma \beta (\delta +\lambda )\left(\frac{\kappa_{1}\varkappa_{1}}{{\text{ν}}_{1}\mu }+\frac{\kappa_{2}\varkappa_{2}}{{\text{ν}}_{2}\mu }+1\right)}-1\right),\Phi^{L}\left(\frac{{\text{ν}}_{1}}{\gamma_{1}}\right)=\frac{{\text{κ}}_{1}{\text{ϰ}}_{1}{\text{γ}}_{2}(\delta +\lambda )({\text{γ}}_{1}\beta \sigma +\eta \alpha {\text{ν}}_{1})}{{\text{γ}}_{1}}\left(\frac{{\text{ν}}_{2}}{\gamma_{2}}-\frac{{\text{ν}}_{1}}{\gamma_{1}}\right),\Phi^{L}\left(\frac{{\text{ν}}_{2}}{\gamma_{2}}\right)=\frac{{\text{κ}}_{2}{\text{ϰ}}_{2}{\text{γ}}_{1}(\delta +\lambda )({\text{γ}}_{2}\beta \sigma +\eta \alpha {\text{ν}}_{2})}{{\text{γ}}_{2}}\left(\frac{{\text{ν}}_{1}}{\gamma_{1}}-\frac{{\text{ν}}_{2}}{\gamma_{2}}\right),\lim_{I\to \infty }\Phi^{L}(I)=\infty .$$

We have $\Phi^{L}(0)<0$ when

(CL) $$\frac{\eta \alpha \rho (\delta +\theta \lambda )}{\mu \sigma \beta (\delta +\lambda )\left(\frac{\kappa_{1}\varkappa_{1}}{{\text{ν}}_{1}\mu }+\frac{\kappa_{2}\varkappa_{2}}{{\text{ν}}_{2}\mu }+1\right)}>1.$$

We note that

$$\frac{{\text{ν}}_{2}}{\gamma_{2}}>\frac{{\text{ν}}_{1}}{\gamma_{1}}⟹\Phi^{L}\left(\frac{{\text{ν}}_{1}}{\gamma_{1}}\right)>0\text{ and }\Phi^{L}\left(\frac{{\text{ν}}_{2}}{\gamma_{2}}\right)<0,\frac{{\text{ν}}_{2}}{\gamma_{2}}<\frac{{\text{ν}}_{1}}{\gamma_{1}}⟹\Phi^{L}\left(\frac{{\text{ν}}_{1}}{\gamma_{1}}\right)<0\text{ and }\Phi^{L}\left(\frac{{\text{ν}}_{2}}{\gamma_{2}}\right)>0.$$

$$\Phi^{L}\left(\min\left\{\frac{{\text{ν}}_{1}}{\gamma_{1}},\frac{{\text{ν}}_{2}}{\gamma_{2}}\right\}\right)>0\text{ and }\Phi^{L}\left(\max\left\{\frac{{\text{ν}}_{1}}{\gamma_{1}},\frac{{\text{ν}}_{2}}{\gamma_{2}}\right\}\right)<0.$$

If condition (CL) is fulfilled, Eq. (37) possesses three positive roots:

$$I^{⁎}\in \left(0,\min\left\{\frac{{\text{ν}}_{1}}{\gamma_{1}},\frac{{\text{ν}}_{2}}{\gamma_{2}}\right\}\right),\overset{¯}{I}\in \left(\min\left\{\frac{{\text{ν}}_{1}}{\gamma_{1}},\frac{{\text{ν}}_{2}}{\gamma_{2}}\right\},\max\left\{\frac{{\text{ν}}_{1}}{\gamma_{1}},\frac{{\text{ν}}_{2}}{\gamma_{2}}\right\}\right),\overset{˜}{I}\in \left(\max\left\{\frac{{\text{ν}}_{1}}{\gamma_{1}},\frac{{\text{ν}}_{2}}{\gamma_{2}}\right\},\infty \right).$$

From Eq. (35), the solution $\overset{¯}{I}$ yields $C_{1}<0$ or $C_{2}<0$, moreover, $\overset{˜}{I}$ gives $C_{1}<0$ and $C_{2}<0$. Thus, the only valid solution is $I^{⁎}$, yielding

$$U^{⁎}=\frac{\rho }{\sigma +\alpha D^{⁎}}>0,\;L^{⁎}=\frac{(1-\theta )\alpha \rho D^{⁎}}{\left(\delta +\lambda )(\sigma +\alpha D^{⁎}\right)}>0,\;D^{⁎}=\frac{\eta I^{⁎}}{\beta }>0,C_{1}^{⁎}=\frac{\varkappa_{1}}{{\text{ν}}_{1}-\gamma_{1}I^{⁎}}>0,\;\;C_{2}^{⁎}=\frac{\varkappa_{2}}{{\text{ν}}_{2}-\gamma_{2}I^{⁎}}>0.$$

The model (22)-(27) produces a basic reproduction number, denoted as $R_{0}^{L}$, with a specific value expressed as

$$R_{0}^{L}=\frac{\eta \alpha \rho (\delta +\theta \lambda )}{\mu \sigma \beta (\delta +\lambda )\left(\frac{\kappa_{1}C_{1}^{0}}{\mu }+\frac{\kappa_{2}C_{2}^{0}}{\mu }+1\right)},$$

and the presence of the infected equilibrium with immunity ${\mathrm{EQ}}^{⁎}(U^{⁎},L^{⁎},I^{⁎},D^{⁎},C_{1}^{⁎},C_{2}^{⁎})$, is established when $R_{0}^{L}>1$. □

### 3.2 Sensitivity analysis

Sensitivity analysis holds a significant role in the study of dynamic systems, specifically in ecological and epidemiological studies [31]. Among the important facets of this study is the sensitivity analysis of model parameters. Specific sensitivity indices for each parameter are provided, illustrating their roles in disease dynamics. A sensitivity analysis of various parameters is presented in this part versus $R_{0}^{L}$.

To conduct sensitivity analysis, we determine the normalized forward sensitivity index of a variable utilizing the formula

(38) $$S_{\omega }=\frac{\omega }{R_{0}^{L}}\frac{\partial R_{0}^{L}}{\partial \omega },$$

which gives the sensitivity index of $R_{0}^{L}$ with respect to the parameter *ω*, and we get sensitivity with respect to $\rho :S_{\rho }=\frac{\rho }{R_{0}^{L}}\frac{\partial R_{0}^{L}}{\partial \rho }=1>0$, and

$$S_{\sigma }=-1<0,\;S_{\alpha }=1>0,\;S_{\mu }=-\frac{\nu_{1}\mu \nu_{2}}{\kappa_{1}\varkappa_{1}\nu_{2}+\kappa_{2}\;\varkappa_{2}\nu_{1}+\nu_{1}\mu \;\nu_{2}}<0,S_{\eta }=1>0,\;S_{\beta }=-1<0,\;S_{\theta }=\frac{\theta \;\lambda }{\theta \;\lambda +\delta }>0,\;S_{\delta }=\frac{\delta \;\lambda \;\left(1-\theta \right)}{\left(\delta +\lambda \right)\left(\theta \;\lambda +\delta \right)}>0,S_{\lambda }=-\frac{\delta \;\lambda \;\left(1-\theta \right)}{\left(\delta +\lambda \right)\left(\theta \;\lambda +\delta \right)}<0,\;S_{\kappa_{1}}=S_{\varkappa_{1}}=-\frac{\kappa_{1}\;\varkappa_{1}\;\nu_{2}}{\kappa_{1}\varkappa_{1}\nu_{2}+\kappa_{2}\varkappa_{2}\nu_{1}+\nu_{1}\mu \nu_{2}}<0,S_{\kappa_{2}}=S_{\varkappa_{2}}=-\frac{\kappa_{2}\varkappa_{2}\nu_{1}}{\kappa_{1}\varkappa_{1}\nu_{2}+\kappa_{2}\varkappa_{2}\nu_{1}+\nu_{1}\mu \nu_{2}}<0,\;S_{\gamma_{1}}=0,\;S_{\gamma_{2}}=0,S_{\nu_{1}}=-S_{\kappa_{1}}>0.\;S_{\nu_{2}}=-S_{\kappa_{2}}>0.$$

It is apparent that parameters $\rho ,\alpha ,\eta ,\theta ,\delta ,\nu_{1}$ and $\nu_{2}$ have positive indices, indicating that $R_{0}^{L}$ will increase as these parameters increase. In addition, the increase or decrease of the two parameters $\gamma_{1}$ and $\gamma_{2}$ has no effect on the value of $R_{0}^{L}$. Other parameters have negative sensitivity indices, indicating that as they increase, $R_{0}^{L}$ decreases.

### 3.3 Global stability

Define a function $\Theta_{j}^{L}(U,L,I,D,C_{1},C_{2})$ and let ${\overset{˜}{\Gamma }}_{j}^{L}$ be the largest invariant subset of $\Gamma_{j}^{L}=\left\{(U,L,I,D,C_{1},C_{2}):\frac{d\Theta_{j}^{L}}{dt}=0\right\}$, j=0,1.

Theorem 3 *(i) In the case that* $R_{0}^{L}\leq 1$ *, the uninfected equilibrium* ${\mathrm{EQ}}^{0}(U^{0},0,0,0,C_{1}^{0},C_{2}^{0})$ *is G.A.S within the set* $O^{L}$ *, and (ii) if* $R_{0}^{L}>1$ *,* ${\mathrm{EQ}}^{0}$ *becomes unstable.*

**Proof.** (i) Define

$$\Theta_{0}^{L}=U^{0}\left(\frac{U}{U^{0}}-1-\ln\left(\frac{U}{U^{0}}\right)\right)+\frac{\delta }{\delta +\theta \lambda }L+\frac{\delta +\lambda }{\delta +\theta \lambda }I+\frac{\alpha U^{0}}{\beta }D+\frac{\kappa_{1}(\delta +\lambda )}{\gamma_{1}(\delta +\theta \lambda )}C_{1}^{0}\left(\frac{C_{1}}{C_{1}^{0}}-1-\ln\left(\frac{C_{1}}{C_{1}^{0}}\right)\right)+\frac{\kappa_{2}(\delta +\lambda )}{\gamma_{2}(\delta +\theta \lambda )}C_{2}^{0}\left(\frac{C_{2}}{C_{2}^{0}}-1-\ln\left(\frac{C_{2}}{C_{2}^{0}}\right)\right).$$

Observe that $\Theta_{0}^{L}(U,L,I,D,C_{1},C_{2})>0$ for all $(U,L,I,D,C_{1},C_{2})>0$ and $\Theta_{0}^{L}(U^{0},0,0,0,C_{1}^{0},C_{2}^{0})=0$. Calculating $\frac{d\Theta_{0}^{L}}{dt}$ along the solutions of (22)-(27) as:

$$\frac{d\Theta_{0}^{L}}{dt}=\left(1-\frac{U^{0}}{U}\right)\left(\rho -\sigma U-\alpha UD\right)+\frac{\delta }{\delta +\theta \lambda }\left((1-\theta )\alpha UD-(\delta +\lambda )L\right)+\frac{\delta +\lambda }{\delta +\theta \lambda }\left(\theta \alpha UD+\delta L-\mu I-\kappa_{1}IC_{1}-\kappa_{2}IC_{2}\right)+\frac{\alpha U^{0}}{\beta }\left(\eta I-\beta D\right)+\frac{\kappa_{1}(\delta +\lambda )}{\gamma_{1}(\delta +\theta \lambda )}\left(1-\frac{C_{1}^{0}}{C_{1}}\right)(\varkappa_{1}+\gamma_{1}IC_{1}-\nu_{1}C_{1})+\frac{\kappa_{2}(\delta +\lambda )}{\gamma_{2}(\delta +\theta \lambda )}\left(1-\frac{C_{2}^{0}}{C_{2}}\right)(\varkappa_{2}+\gamma_{2}IC_{2}-\nu_{2}C_{2})=\left(1-\frac{U^{0}}{U}\right)\left(\rho -\sigma U\right)-\frac{\delta +\lambda }{\delta +\theta \lambda }\mu I+\frac{\alpha U^{0}}{\beta }\eta I+\frac{\kappa_{1}(\delta +\lambda )}{\gamma_{1}(\delta +\theta \lambda )}\left(1-\frac{C_{1}^{0}}{C_{1}}\right)(\varkappa_{1}-\nu_{1}C_{1})-\frac{\kappa_{1}(\delta +\lambda )}{\delta +\theta \lambda }C_{1}^{0}I+\frac{\kappa_{2}(\delta +\lambda )}{\gamma_{2}(\delta +\theta \lambda )}\left(1-\frac{C_{2}^{0}}{C_{2}}\right)(\varkappa_{2}-\nu_{2}C_{2})-\frac{\kappa_{2}(\delta +\lambda )}{\delta +\theta \lambda }C_{2}^{0}I.$$

Using $\rho =\sigma U^{0}$, $\varkappa_{1}=\nu_{1}C_{1}^{0}$ and $\varkappa_{2}=\nu_{2}C_{2}^{0}$ we obtain

$$\frac{d\Theta_{0}^{L}}{dt}=-{\frac{\sigma \left(U-U^{0}\right)}{U}}^{2}-\frac{\kappa_{1}{\text{ν}}_{1}(\delta +\lambda )}{\gamma_{1}(\delta +\theta \lambda )}\frac{{\left(C_{1}-C_{1}^{0}\right)}^{2}}{C_{1}}-\frac{\kappa_{2}{\text{ν}}_{2}(\delta +\lambda )}{\gamma_{2}(\delta +\theta \lambda )}\frac{{\left(C_{2}-C_{2}^{0}\right)}^{2}}{C_{2}}+\left(\frac{\alpha U^{0}}{\beta }\eta -\frac{\mu (\delta +\lambda )}{\left(\delta +\theta \lambda \right)}-\frac{\kappa_{1}(\delta +\lambda )C_{1}^{0}}{\delta +\theta \lambda }-\frac{\kappa_{2}(\delta +\lambda )C_{2}^{0}}{\delta +\theta \lambda }\right)I=-{\frac{\sigma \left(U-U^{0}\right)}{U}}^{2}-\frac{\kappa_{1}{\text{ν}}_{1}(\delta +\lambda )}{\gamma_{1}(\delta +\theta \lambda )}\frac{{\left(C_{1}-C_{1}^{0}\right)}^{2}}{C_{1}}-\frac{\kappa_{2}{\text{ν}}_{2}(\delta +\lambda )}{\gamma_{2}(\delta +\theta \lambda )}\frac{{\left(C_{2}-C_{2}^{0}\right)}^{2}}{C_{2}}+\frac{\eta \alpha \rho }{\beta \sigma R_{0}^{L}}\left(R_{0}^{L}-1\right)I.$$

Therefore, if $R_{0}^{L}\leq 1$, then $\frac{d\Theta_{0}^{L}}{dt}\leq 0$ for all $\left(U,L,I,D,C_{1},C_{2})\in (0,\infty \right)$. Moreover, $\frac{d\Theta_{0}^{L}}{dt}=0$ when $U(t)=U^{0}$, $C_{1}(t)=C_{1}^{0}$, $C_{2}(t)=C_{2}^{0}$ and D(t)=0 for all *t*.

The solutions to the system (22)-(27) demonstrate convergence towards ${\overset{˜}{\Gamma }}_{0}^{L}$ which comprises elements that meet the conditions $U(t)=U^{0}$, $C_{1}(t)=C_{1}^{0}$, $C_{2}(t)=C_{2}^{0}$ and I(t)=0. It follows from Eq. (22) that

$$0=\frac{dU}{dt}=\rho -\sigma U_{0}-\alpha U_{0}D⟹D(t)=0.$$

Further, from Eq. (24) we have

$$0=\frac{dI(t)}{dt}=\delta L⟹L(t)=0.$$

Thus, ${\overset{˜}{\Gamma }}_{0}^{L}=\{{\mathrm{EQ}}^{0}\}$ and the LaSalle-Lyapunov theorem implies that ${\mathrm{EQ}}^{0}$ is G.A.S in $O^{L}$.

(ii) The Jacobian matrix $J_{2}=J_{2}(U,L,I,D,C_{1},C_{2})$ of system (22)-(27) is calculated as:

$$J_{2}=\left(\begin{array}{cccccc} -\alpha D-\sigma \; & 0\; & 0\; & -\alpha U\; & 0\; & 0\\ \alpha (1-\theta )D\; & -\delta -\lambda \; & 0\; & \alpha (1-\theta )U\; & 0\; & 0\\ \theta \alpha D\; & \delta \; & -{\text{κ}}_{1}C_{1}-{\text{κ}}_{2}C_{2}-\mu \; & \theta \alpha U\; & -{\text{κ}}_{1}I\; & -{\text{κ}}_{2}I\\ 0\; & 0\; & \eta \; & -\beta \; & 0\; & 0\\ 0\; & 0\; & {\text{γ}}_{1}C_{1}\; & 0\; & {\text{γ}}_{1}I-{\text{ν}}_{1}\; & 0\\ 0\; & 0\; & {\text{γ}}_{2}C_{2}\; & 0\; & 0\; & {\text{γ}}_{2}I-{\text{ν}}_{2} \end{array}\right).$$

Then, the characteristic equation at the equilibrium ${\mathrm{EQ}}^{0}$ is expressed as

(39) $$\det(J_{2}-\xi I)=(\xi +\sigma )(\xi +\nu_{1})(\xi +\nu_{2})\left(\xi^{3}+P_{2}^{L}\xi^{2}+P_{1}^{L}\xi +P_{0}^{L}\right)=0,$$

where *ξ* is the eigenvalue and

$$P_{2}^{L}=\mu +\delta +\beta +\lambda +\frac{\kappa_{1}\varkappa_{1}}{{\text{ν}}_{1}}+\frac{\kappa_{2}\varkappa_{2}}{{\text{ν}}_{2}},P_{1}^{L}=-\frac{\eta \theta \alpha \rho }{\sigma }+\beta (\delta +\lambda )+\frac{\left(\delta +\beta +\lambda )({\text{κ}}_{2}{\text{ϰ}}_{2}{\text{ν}}_{1}+{\text{κ}}_{1}{\text{ϰ}}_{1}{\text{ν}}_{2}+\mu {\text{ν}}_{1}{\text{ν}}_{2}\right)}{{\text{ν}}_{1}{\text{ν}}_{2}},P_{0}^{L}=\frac{{\text{κ}}_{2}{\text{ϰ}}_{2}\beta \sigma (\delta +\lambda ){\text{ν}}_{1}+({\text{κ}}_{1}{\text{ϰ}}_{1}\beta \sigma (\delta +\lambda )+(\mu \beta \sigma (\delta +\lambda )-\eta \alpha \rho (\delta +\theta \lambda )){\text{ν}}_{1}){\text{ν}}_{2}}{\sigma {\text{ν}}_{1}{\text{ν}}_{2}},=\frac{\beta (\delta +\lambda )(\kappa_{2}\varkappa_{2}{\text{ν}}_{1}+\kappa_{1}\varkappa_{1}{\text{ν}}_{2}+\beta {\text{ν}}_{1}{\text{ν}}_{2})}{{\text{ν}}_{1}{\text{ν}}_{2}}(1-R_{0}^{L}).$$

Clearly if $R_{0}^{L}>1$, then $P_{0}^{L}<0$ and Eq. (39) has a positive root and hence ${\mathrm{EQ}}^{0}$ is unstable. □

Theorem 4 *The infected equilibrium* ${\mathrm{EQ}}^{⁎}(U^{⁎},L^{⁎},I^{⁎},D^{⁎},C_{1}^{⁎},C_{2}^{⁎})$ *of system* (22) *-* (27) *is G.A.S in* ${\overset{˚}{O}}^{L}$ *if* $R_{0}>1$ *.*

**Proof.** Let

$$\Theta_{1}^{L}=U^{⁎}\left(\frac{U}{U^{⁎}}-1-\ln\left(\frac{U}{U^{⁎}}\right)\right)+\frac{\delta }{\delta +\theta \lambda }L^{⁎}\left(\frac{L}{L^{⁎}}-1-\ln\left(\frac{L}{L^{⁎}}\right)\right)+\frac{\delta +\lambda }{\delta +\theta \lambda }I^{⁎}\left(\frac{I}{I^{⁎}}-1-\ln\left(\frac{I}{I^{⁎}}\right)\right)+\frac{\alpha U^{⁎}}{\beta }D^{⁎}\left(\frac{D}{D^{⁎}}-1-\ln\left(\frac{D}{D^{⁎}}\right)\right)+\frac{\kappa_{1}(\delta +\lambda )}{\gamma_{1}(\delta +\theta \lambda )}C_{1}^{⁎}\left(\frac{C_{1}}{C_{1}^{⁎}}-1-\ln\left(\frac{C_{1}}{C_{1}^{⁎}}\right)\right)+\frac{\kappa_{2}(\delta +\lambda )}{\gamma_{2}(\delta +\theta \lambda )}C_{2}^{⁎}\left(\frac{C_{2}}{C_{2}^{⁎}}-1-\ln\left(\frac{C_{2}}{C_{2}^{⁎}}\right)\right).$$

Calculating $\frac{d\Theta_{1}^{L}}{dt}$ along the trajectories of (22)-(27):

$$\frac{d\Theta_{1}^{L}}{dt}=\left(1-\frac{U^{⁎}}{U}\right)\left(\rho -\sigma U-\alpha UD\right)+\frac{\delta }{\delta +\theta \lambda }\left(1-\frac{L^{⁎}}{L}\right)\left((1-\theta )\alpha UD-(\delta +\lambda )L\right)+\frac{\delta +\lambda }{\delta +\theta \lambda }\left(1-\frac{I^{⁎}}{I}\right)\left(\theta \alpha UD+\delta L-\mu I-\kappa_{1}IC_{1}-\kappa_{2}IC_{2}\right)+\frac{\alpha U^{⁎}}{\beta }\left(1-\frac{D^{⁎}}{D}\right)\left(\eta I-\beta D\right)+\frac{\kappa_{1}(\delta +\lambda )}{\gamma_{1}(\delta +\theta \lambda )}\left(1-\frac{C_{1}^{⁎}}{C_{1}}\right)(\varkappa_{1}+\gamma_{1}IC_{1}-\nu_{1}C_{1})+\frac{\kappa_{2}(\delta +\lambda )}{\gamma_{2}(\delta +\theta \lambda )}\left(1-\frac{C_{2}^{⁎}}{C_{2}}\right)(\varkappa_{2}+\gamma_{2}IC_{2}-\nu_{2}C_{2})=\left(1-\frac{U^{⁎}}{U}\right)(\rho -\sigma U)-\frac{\delta }{\delta +\theta \lambda }\frac{(1-\theta )\alpha UDL^{⁎}}{L}+\frac{\delta (\delta +\lambda )}{\delta +\theta \lambda }L^{⁎}-\frac{\delta +\lambda }{\delta +\theta \lambda }\mu I-\frac{\delta +\lambda }{\delta +\theta \lambda }\frac{\theta \alpha UDI^{⁎}}{I}-\frac{\delta (\delta +\lambda )}{\delta +\theta \lambda }\frac{I^{⁎}L}{I}+\frac{\delta +\lambda }{\delta +\theta \lambda }\mu I^{⁎}+\frac{\kappa_{1}(\delta +\lambda )}{\left(\delta +\theta \lambda \right)}I^{⁎}C_{1}+\frac{\kappa_{2}(\delta +\lambda )}{\left(\delta +\theta \lambda \right)}I^{⁎}C_{2}+\frac{\alpha U^{⁎}}{\beta }\eta I-\frac{\alpha \eta U^{⁎}}{\beta }\frac{D^{⁎}I}{D}+\alpha U^{⁎}D^{⁎}+\frac{\kappa_{1}(\delta +\lambda )}{\gamma_{1}(\delta +\theta \lambda )}\left(1-\frac{C_{1}^{⁎}}{C_{1}}\right)(\varkappa_{1}-\nu_{1}C_{1})-\frac{\kappa_{1}(\delta +\lambda )}{\left(\delta +\theta \lambda \right)}C_{1}^{⁎}I+\frac{\kappa_{2}(\delta +\lambda )}{\gamma_{2}(\delta +\theta \lambda )}\left(1-\frac{C_{2}^{⁎}}{C_{2}}\right)(\varkappa_{2}-\nu_{2}C_{2})-\frac{\kappa_{2}(\delta +\lambda )}{\left(\delta +\theta \lambda \right)}C_{2}^{⁎}I.$$

The equilibrium conditions of ${\mathrm{EQ}}^{⁎}$ imply

$$\rho =\sigma U^{⁎}+\alpha U^{⁎}D^{⁎},\frac{\delta (\delta +\lambda )}{\delta +\theta \lambda }L^{⁎}=\frac{(1-\theta )\delta }{\delta +\theta \lambda }\alpha U^{⁎}D^{⁎}\;\frac{\delta +\lambda }{\delta +\theta \lambda }\mu I^{⁎}=\alpha U^{⁎}D^{⁎}-\frac{\delta +\lambda }{\delta +\theta \lambda }\kappa_{1}I^{⁎}C_{1}^{⁎}-\frac{\delta +\lambda }{\delta +\theta \lambda }\kappa_{2}I^{⁎}C_{2}^{⁎},\;\;\eta I^{⁎}=\beta D^{⁎},\varkappa_{1}=\nu_{1}C_{1}^{⁎}-\gamma_{1}I^{⁎}C_{1}^{⁎},\varkappa_{2}=\nu_{2}C_{2}^{⁎}-\gamma_{2}I^{⁎}C_{2}^{⁎}.$$

Then, we get

$$\frac{d\Theta_{1}^{L}}{dt}=\left(1-\frac{U^{⁎}}{U}\right)(\sigma U^{⁎}+\alpha U^{⁎}D^{⁎}-\sigma U)-\frac{\delta (1-\theta )}{\delta +\theta \lambda }\alpha U^{⁎}D^{⁎}\frac{UDL^{⁎}}{U^{⁎}D^{⁎}L}+\frac{(1-\theta )\delta }{\delta +\theta \lambda }\alpha U^{⁎}D^{⁎}-\frac{\delta +\lambda }{\delta +\theta \lambda }\mu I-\frac{(\delta +\lambda )\theta }{\delta +\theta \lambda }\alpha U^{⁎}D^{⁎}\frac{UDI^{⁎}}{U^{⁎}D^{⁎}I}-\frac{(1-\theta )\delta }{\delta +\theta \lambda }\alpha U^{⁎}D^{⁎}\frac{I^{⁎}L}{IL^{⁎}}+\alpha U^{⁎}D^{⁎}-\frac{\delta +\lambda }{\delta +\theta \lambda }\kappa_{1}I^{⁎}C_{1}^{⁎}-\frac{\delta +\lambda }{\delta +\theta \lambda }\kappa_{2}I^{⁎}C_{2}^{⁎}+\frac{\kappa_{1}(\delta +\lambda )}{\left(\delta +\theta \lambda \right)}I^{⁎}C_{1}+\frac{\kappa_{2}(\delta +\lambda )}{\left(\delta +\theta \lambda \right)}I^{⁎}C_{2}+\frac{\alpha U^{⁎}}{\beta }\eta I-\alpha U^{⁎}D^{⁎}\frac{D^{⁎}I}{DI^{⁎}}+\alpha U^{⁎}D^{⁎}+\frac{\kappa_{1}(\delta +\lambda )}{\gamma_{1}(\delta +\theta \lambda )}\left(1-\frac{C_{1}^{⁎}}{C_{1}}\right)(\nu_{1}C_{1}^{⁎}-\gamma_{1}I^{⁎}C_{1}^{⁎}-\nu_{1}C_{1})-\frac{\kappa_{1}(\delta +\lambda )}{\left(\delta +\theta \lambda \right)}C_{1}^{⁎}I+\frac{\kappa_{2}(\delta +\lambda )}{\gamma_{2}(\delta +\theta \lambda )}\left(1-\frac{C_{2}^{⁎}}{C_{2}}\right)(\nu_{2}C_{2}^{⁎}-\gamma_{2}I^{⁎}C_{2}^{⁎}-\nu_{2}C_{2})-\frac{\kappa_{2}(\delta +\lambda )}{\left(\delta +\theta \lambda \right)}C_{2}^{⁎}I.$$

It follows that

$$\frac{d\Theta_{1}^{L}}{dt}=\left(1-\frac{U^{⁎}}{U}\right)(\sigma U^{⁎}-\sigma U)+\alpha U^{⁎}D^{⁎}\left(1-\frac{U^{⁎}}{U}\right)-\frac{\delta (1-\theta )}{\delta +\theta \lambda }\alpha U^{⁎}D^{⁎}\frac{UDL^{⁎}}{U^{⁎}D^{⁎}L}+\frac{(1-\theta )\delta }{\delta +\theta \lambda }\alpha U^{⁎}D^{⁎}-\frac{(\delta +\lambda )\theta }{\delta +\theta \lambda }\alpha U^{⁎}D^{⁎}\frac{UDI^{⁎}}{U^{⁎}D^{⁎}I}-\frac{(1-\theta )\delta }{\delta +\theta \lambda }\alpha U^{⁎}D^{⁎}\frac{I^{⁎}L}{IL^{⁎}}+2\alpha U^{⁎}D^{⁎}-\frac{\delta +\lambda }{\delta +\theta \lambda }\kappa_{1}I^{⁎}C_{1}^{⁎}-\frac{\delta +\lambda }{\delta +\theta \lambda }\kappa_{2}I^{⁎}C_{2}^{⁎}+\frac{\kappa_{1}(\delta +\lambda )}{\left(\delta +\theta \lambda \right)}I^{⁎}C_{1}+\frac{\kappa_{2}(\delta +\lambda )}{\left(\delta +\theta \lambda \right)}I^{⁎}C_{2}-\alpha U^{⁎}D^{⁎}\frac{D^{⁎}I}{DI^{⁎}}+\frac{\kappa_{1}(\delta +\lambda )}{\gamma_{1}(\delta +\theta \lambda )}\left(1-\frac{C_{1}^{⁎}}{C_{1}}\right)(\nu_{1}C_{1}^{⁎}-\nu_{1}C_{1})-\frac{\kappa_{1}(\delta +\lambda )}{\left(\delta +\theta \lambda \right)}I^{⁎}C_{1}^{⁎}\left(1-\frac{C_{1}^{⁎}}{C_{1}}\right)+\frac{\kappa_{2}(\delta +\lambda )}{\gamma_{2}(\delta +\theta \lambda )}\left(1-\frac{C_{2}^{⁎}}{C_{2}}\right)(\nu_{2}C_{2}^{⁎}-\nu_{2}C_{2})-\frac{\kappa_{2}(\delta +\lambda )}{\left(\delta +\theta \lambda \right)}I^{⁎}C_{2}^{⁎}\left(1-\frac{C_{2}^{⁎}}{C_{2}}\right)+\left(-\frac{\delta +\lambda }{\delta +\theta \lambda }\mu I^{⁎}+\alpha U^{⁎}D^{⁎}-\frac{\kappa_{1}(\delta +\lambda )}{\left(\delta +\theta \lambda \right)}I^{⁎}C_{1}^{⁎}-\frac{\kappa_{2}(\delta +\lambda )}{\left(\delta +\theta \lambda \right)}I^{⁎}C_{2}^{⁎}\right)\frac{I}{I^{⁎}}.$$

We have

$$\left(-\frac{\delta +\lambda }{\delta +\theta \lambda }\mu I^{⁎}+\alpha U^{⁎}D^{⁎}-\frac{\kappa_{1}(\delta +\lambda )}{\left(\delta +\theta \lambda \right)}I^{⁎}C_{1}^{⁎}-\frac{\kappa_{2}(\delta +\lambda )}{\left(\delta +\theta \lambda \right)}I^{⁎}C_{2}^{⁎}\right)\frac{I}{I^{⁎}}=0.$$

Then

$$\frac{d\Theta_{1}^{L}}{dt}=-\frac{\sigma {\left(U-U^{⁎}\right)}^{2}}{U}+\frac{\delta (1-\theta )}{\delta +\theta \lambda }\alpha U^{⁎}D^{⁎}\left[4-\frac{U^{⁎}}{U}-\frac{UDL^{⁎}}{U^{⁎}D^{⁎}L}-\frac{I^{⁎}L}{IL^{⁎}}-\frac{D^{⁎}I}{DI^{⁎}}\right]+\frac{(\delta +\lambda )\theta }{\delta +\theta \lambda }\alpha U^{⁎}D^{⁎}\left[3-\frac{U^{⁎}}{U}-\frac{UDI^{⁎}}{U^{⁎}D^{⁎}I}-\frac{D^{⁎}I}{DI^{⁎}}\right]-\frac{\kappa_{1}(\delta +\lambda )}{\delta +\theta \lambda }I^{⁎}C_{1}^{⁎}\left[2-\frac{C_{1}^{⁎}}{C_{1}}-\frac{C_{1}}{C_{1}^{⁎}}\right]-\frac{\kappa_{2}(\delta +\lambda )}{\delta +\theta \lambda }I^{⁎}C_{2}^{⁎}\left[2-\frac{C_{2}^{⁎}}{C_{2}}-\frac{C_{2}}{C_{2}^{⁎}}\right]-\frac{\kappa_{1}{\text{ν}}_{1}(\delta +\lambda )}{\gamma_{1}(\delta +\theta \lambda )}\frac{{\left(C_{1}-C_{1}^{⁎}\right)}^{2}}{C_{1}}-\frac{\kappa_{2}{\text{ν}}_{2}(\delta +\lambda )}{\gamma_{1}(\delta +\theta \lambda )}\frac{{\left(C_{2}-C_{2}^{⁎}\right)}^{2}}{C_{2}}.$$

The equilibrium conditions give $I^{⁎}-\frac{{\text{ν}}_{1}}{\gamma_{1}}=-\frac{\varkappa_{1}}{\gamma_{1}C_{1}^{⁎}}$ and $I^{⁎}-\frac{{\text{ν}}_{2}}{\gamma_{2}}=-\frac{\varkappa_{2}}{\gamma_{2}C_{2}^{⁎}}$. Hence

$$\frac{d\Theta_{1}^{L}}{dt}=-\frac{\sigma {\left(U-U^{⁎}\right)}^{2}}{U}-\frac{\kappa_{1}\varkappa_{1}(\delta +\lambda )}{\gamma_{1}(\delta +\theta \lambda )}\frac{{\left(C_{1}-C_{1}^{⁎}\right)}^{2}}{C_{1}C_{1}^{⁎}}-\frac{\kappa_{2}\varkappa_{2}(\delta +\lambda )}{\gamma_{2}(\delta +\theta \lambda )}\frac{{\left(C_{2}-C_{2}^{⁎}\right)}^{2}}{C_{2}C_{2}^{⁎}}+\frac{(1-\theta )\delta }{\delta +\theta \lambda }\alpha U^{⁎}D^{⁎}\left[4-\frac{U^{⁎}}{U}-\frac{UDL^{⁎}}{U^{⁎}D^{⁎}L}-\frac{I^{⁎}L}{IL^{⁎}}-\frac{ID^{⁎}}{I^{⁎}D}\right]+\frac{\theta (\delta +\lambda )}{\delta +\theta \lambda }\alpha U^{⁎}D^{⁎}\left[3-\frac{U^{⁎}}{U}-\frac{UDI^{⁎}}{U^{⁎}D^{⁎}I}-\frac{ID^{⁎}}{I^{⁎}D}\right].$$

From inequality (20) we obtain $\frac{d\Theta_{1}^{L}}{dt}\leq 0$ for all $U,L,I,D,C_{1},C_{2}>0$. Further, $\frac{d\Theta_{1}^{L}}{dt}=0$ when $\left(U,L,I,D,C_{1},C_{2})=(U^{⁎},L^{⁎},I^{⁎},D^{⁎},C_{1}^{⁎},C_{2}^{⁎}\right)$. This gives ${\overset{˜}{\Gamma }}_{1}^{L}=\{{\mathrm{EQ}}^{⁎}\}$ and according to L-LAS theorem, ${\mathrm{EQ}}^{⁎}$ is G.A.S in ${\overset{˚}{O}}^{L}$. □

## 4 Numerical simulations

In this section, we illustrate our theoretical findings for models (6)-(10) and (22)-(27) by conducting some numerical computations. To get the numerical solutions of the models, we utilize MATLAB's ODE45 solver.

### 4.1 Numerical simulations for model (6)-(10)

Within this subsection, we conduct numerical simulations to validate the global stability of equilibria for system (6)-(10), employing the values specified in Table 1.

**Table 1:** The parameter values for model (6)-(10).

Parameter	Value	Source	Value	Parameter	Source
*ρ*	10^7^	[16]	*γ*_1_	4.44 × 10^−4^	[7]
*σ*	0.14	[7], [16]	*γ*_2_	1.53 × 10^−3^	[7]
*α*	Varied		*ν*_1_	0.5	[7], [33]
*μ*	0.14	[7], [16]	*ν*_2_	0.5	[7], [33]
*η*	10^4^	[32], [7], [16]	*ϰ*_1_	30	[7], [33]
*β*	3.48	[7], [16]	*κ*_2_	1.04 × 10^−5^	[7]
*κ*_1_	2.77 × 10^−6^	[7]	*ϰ*_2_	30	[7], [33]

#### 4.1.1 Stability of equilibria

To demonstrate that the trajectories of the system with any starting point converge to an equilibrium point, the following various initial conditions are employed:

**IS-1:**$\left(U,I,D,C_{1},C_{2})(0)=(6\times 10^{7},0.5,150,90,90\right)$,

**IS-2:**$\left(U,I,D,C_{1},C_{2})(0)=(4\times 10^{7},1,357,60,60\right)$,

**IS-3:**$\left(U,I,D,C_{1},C_{2})(0)=(2\times 10^{7},3,500,30,30\right)$.

**Figure 1:**
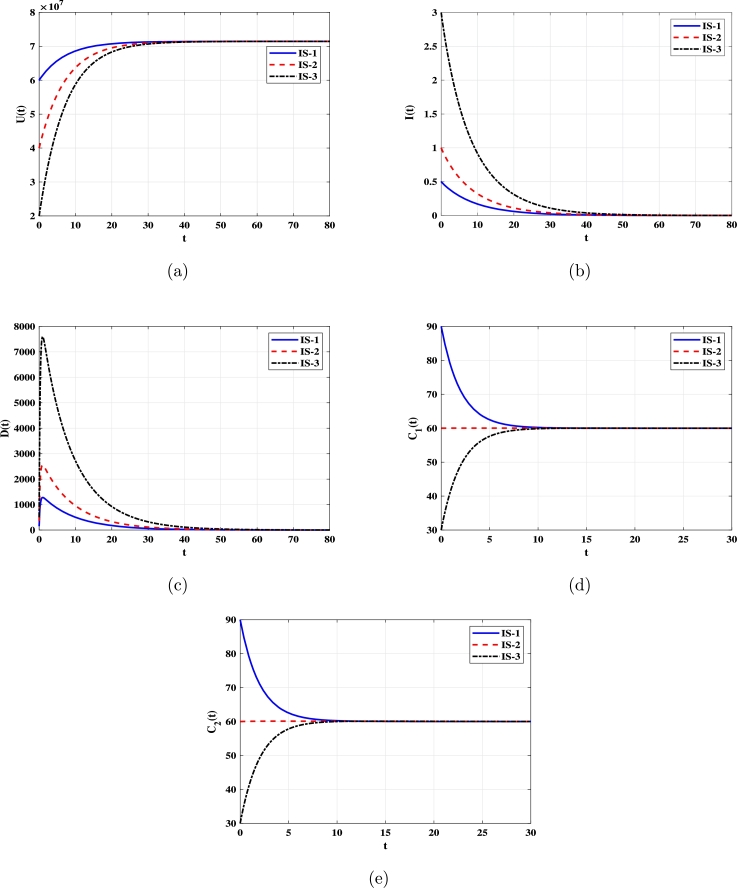
Solutions of system (6)-(10) with various initial conditions converge to ${\mathrm{EQ}}^{0}=\left(7.143\times 10^{7},0,0,60,60\right)$ when *R*_0_ ≤ 1. Panels depict: (a) Uninfected monocytes. (b) Infected monocytes. (c) DENV. (d) Non-specific CTLs. (e) Strain-specific CTLs.

Choosing different values of *α* with the given initial conditions results in the following scenarios:

***Case 1 (Stability of***${\mathrm{EQ}}^{0}$***)***: Let $\alpha =1.72\times 10^{-13}$. In this instance, $R_{0}=0.25<1$. As depicted in Figs. 1a–1e, solutions with initial conditions IS-1, IS-2, and IS-3 converge to the uninfected equilibrium ${\mathrm{EQ}}^{0}=\left(7.143\times 10^{7},0,0,60,60\right)$. According to Theorem 1, ${\mathrm{EQ}}^{0}$ is G.A.S. In this case, DENV will perish, and the number of uninfected monocytes will return to its normal level.

**Figure 2:**
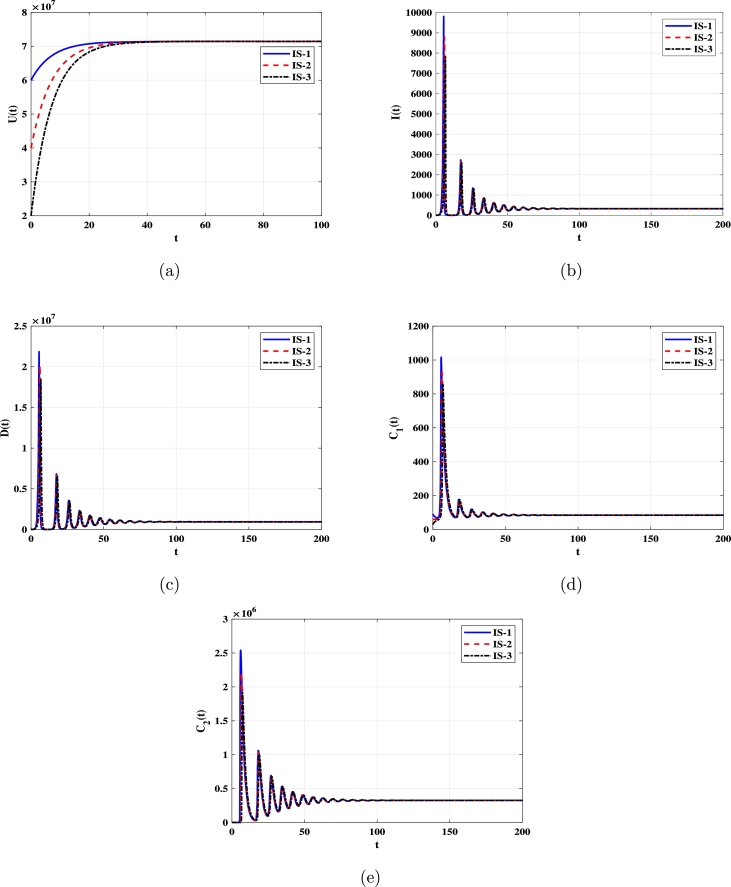
Solutions of system (6)-(10) with various initial conditions converge to ${\mathrm{EQ}}^{⁎}=\left(7.142\times 10^{7},326.73,938900,84.52,325936\right)$ when *R*_0_ > 1. Panels depict: (a) Uninfected monocytes. (b) Infected monocytes. (c) DENV. (d) Non-specific CTLs. (e) Strain-specific CTLs.

***Case 2 (Stability of***${\mathrm{EQ}}^{⁎}$***)***: Selecting $\alpha =1.72\times 10^{-11}$ yields $R_{0}=25>1$. Figs. 2a–2e illustrate that solutions starting with initial conditions IS-1, IS-2, and IS-3 approach the infected equilibrium ${\mathrm{EQ}}^{⁎}=\left(7.142\times 10^{7},326.74,938900,84.52,325936\right)$. As per Lemma 2 and Theorem 2, ${\mathrm{EQ}}^{⁎}$ exists and is G.A.S.

### 4.2 Numerical simulations for model (22)-(27)

We will conduct numerical simulations for the system (22)-(27). The parameter values from Table 1 are considered, along with additional parameters: θ=0.3, δ=0.9 and λ=0.14.

#### 4.2.1 Sensitivity analysis of $R_{0}^{L}$ to the parameters for model (22)-(27)

**Figure 3:**
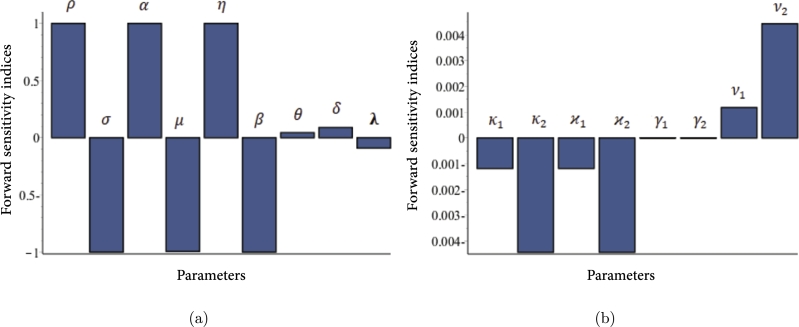
The sensitivities of the model parameters that influence the basic reproduction number $R_{0}^{L}$ of the system (22)-(27). The parameters in Figure (a) exhibit sensitivity indexes higher than those in Figure (b).

**Figure 4:**
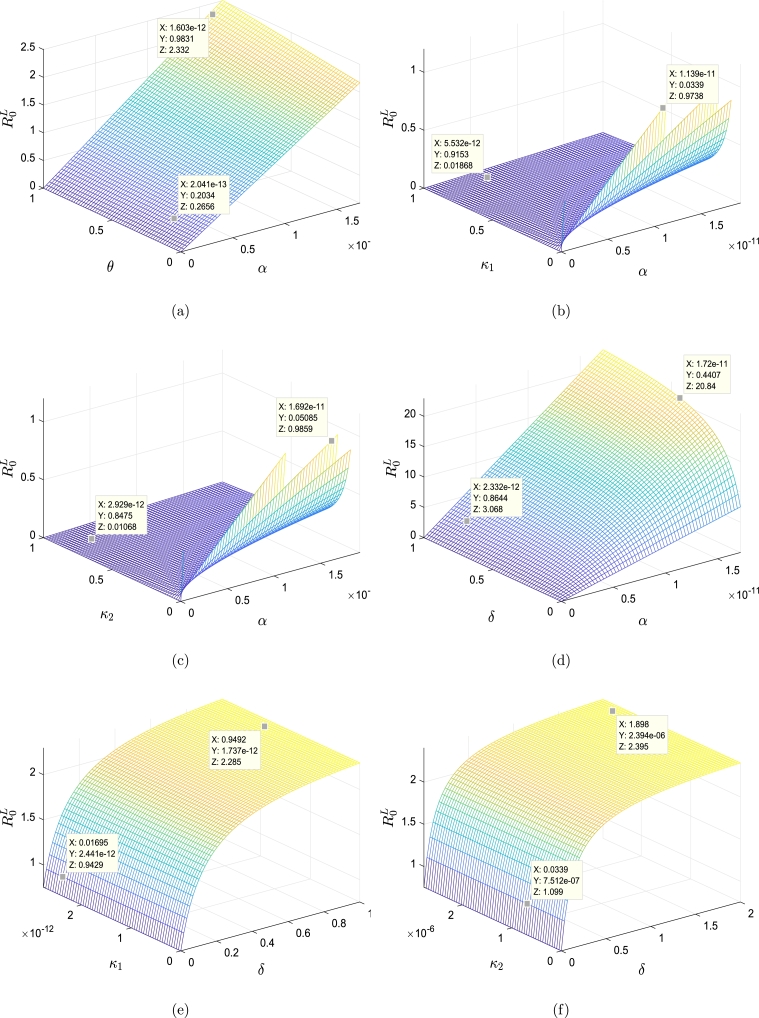

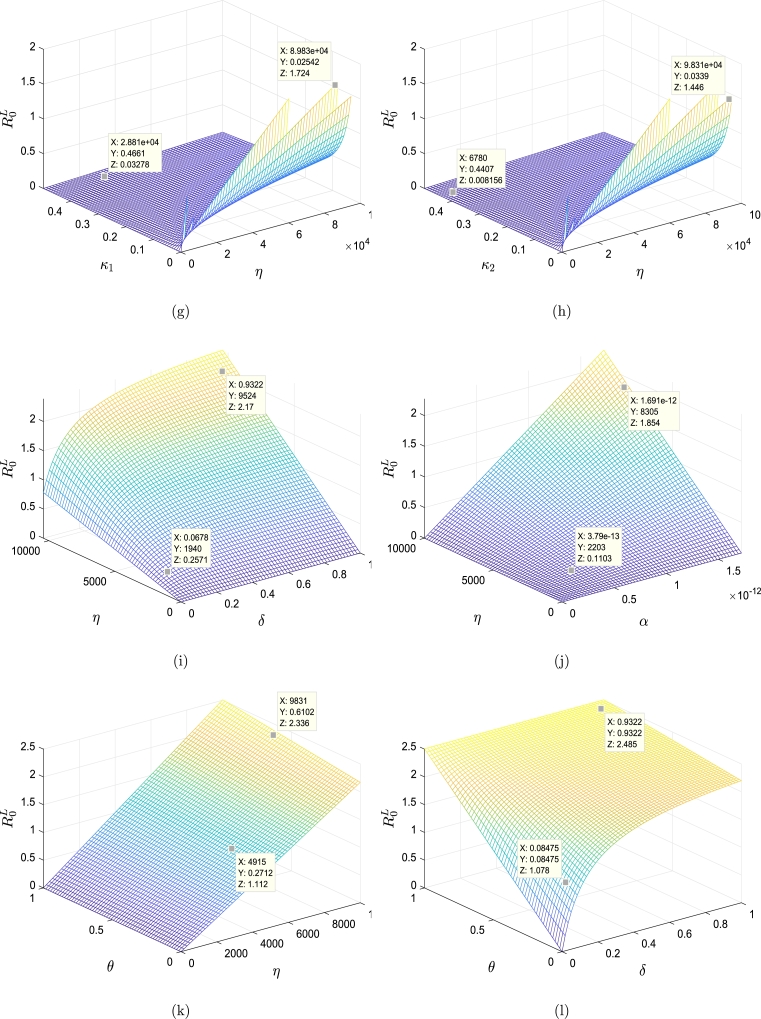

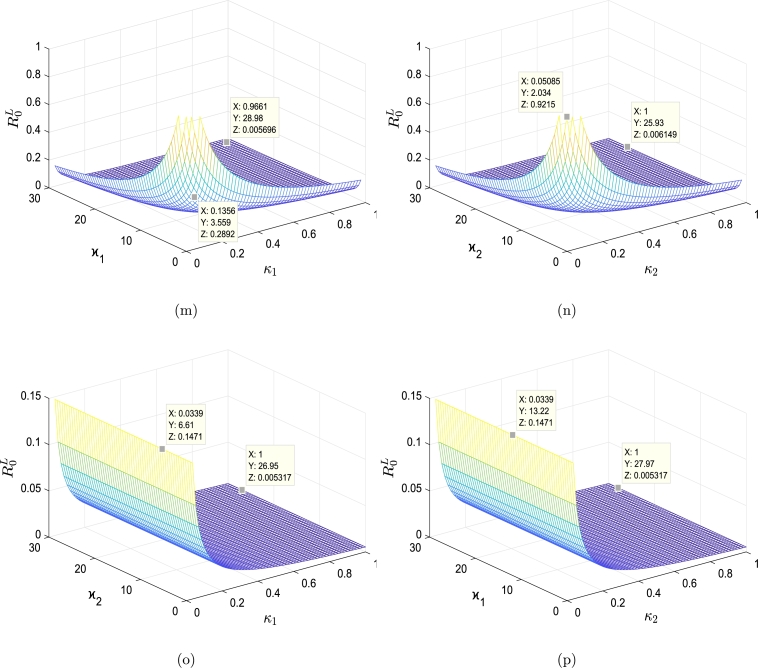
Impact of various parameters for model (22)-(27) on behavior of the basic reproduction number $R_{0}^{L}$. (a) $R_{0}^{L}$ versus *α* and *θ*. (b) $R_{0}^{L}$ versus *α* and *κ*_1_. (c) $R_{0}^{L}$ versus *α* and *κ*_2_. (d) $R_{0}^{L}$ versus *α* and *δ*. (e) $R_{0}^{L}$ versus *δ* and *κ*_1_. (f) $R_{0}^{L}$ versus *δ* and *κ*_2_. (g) $R_{0}^{L}$ versus *η* and *κ*_1_. (h) $R_{0}^{L}$ versus *η* and *κ*_2_. (i) $R_{0}^{L}$ versus *δ* and *η*. (j) $R_{0}^{L}$ versus *α* and *η*. (k) $R_{0}^{L}$ versus *η* and *θ*. (l) $R_{0}^{L}$ versus *δ* and *θ*. (m) $R_{0}^{L}$ versus the parameters *κ*_1_ and *ϰ*_1_. (n) $R_{0}^{L}$ versus *κ*_2_ and *ϰ*_2_. (o) $R_{0}^{L}$ versus *κ*_1_ and *ϰ*_2_. (p) $R_{0}^{L}$ versus *κ*_2_ and *ϰ*_1_.

With pick $\alpha =1.72\times 10^{-12}$, the derived sensitivity indices of different model parameters calculated using (38) are shown in Figs. 3a–3b and Table 2. From Table 2, we find that a 10% increase (or decrease) in the value of $\rho ,\alpha ,\eta ,\theta ,\delta ,\nu_{1}$ and $\nu_{2}$ increases (or decreases) $R_{0}^{L}$ by 10%,10%,10%, 0.446%,0.90%,0.0118% and 0.0443%, respectively. A 10% increase in the values of $\sigma ,\mu ,\beta ,\kappa_{1},\kappa_{2},\varkappa_{1},\varkappa_{2}$ and *λ*, on the other hand, reduces $R_{0}^{L}$ by 10%,9.94%,10%,0.0118%,0.0443%,0.0118%,0.0443% and 0.9%, respectively. We presented the influence of selected vital parameters on $R_{0}^{L}$ graphically in Figs. 4a-4p. It is important to note that, $R_{0}^{L}$ has remained relatively low with the increasing rates of the killing of infected cells by CTLs and rates of production CTLs, which indicates that CTLs play an important role in DENV infection control.

**Table 2:** Sensitivity index of $R_{0}^{L}$.

Parameter	Sensitivity index	Parameter	Sensitivity index	Parameter	Sensitivity index
*ρ*	1	*β*	−1	*γ*_1_	0
*σ*	−1	*κ*_1_	−0.118 × 10^−2^	*γ*_2_	0
*α*	1	*κ*_2_	−0.443 × 10^−2^	*ν*_1_	0.118 × 10^−2^
*μ*	−0.994	*ϰ*_1_	−0.118 × 10^−2^	*ν*_2_	0.443 × 10^−2^
*η*	1	*ϰ*_2_	−0.443 × 10^−2^	*λ*	−0.9 × 10^−1^
*θ*	0.446 × 10^−1^	*δ*	0.90 × 10^−1^

#### 4.2.2 Stability of equilibria

Initially, we consider three distinct initial states:

**IS-1:**$\left(U,L,I,D,C_{1},C_{2})(0)=(6\times 10^{7},700,0.5,150,90,90\right)$,

**IS-2:**$\left(U,L,I,D,C_{1},C_{2})(0)=(4\times 10^{7},500,1,357,60,60\right)$,

**IS-3:**$\left(U,L,I,D,C_{1},C_{2})(0)=(2\times 10^{7},200,3,500,30,30\right)$.

**Figure 5:**
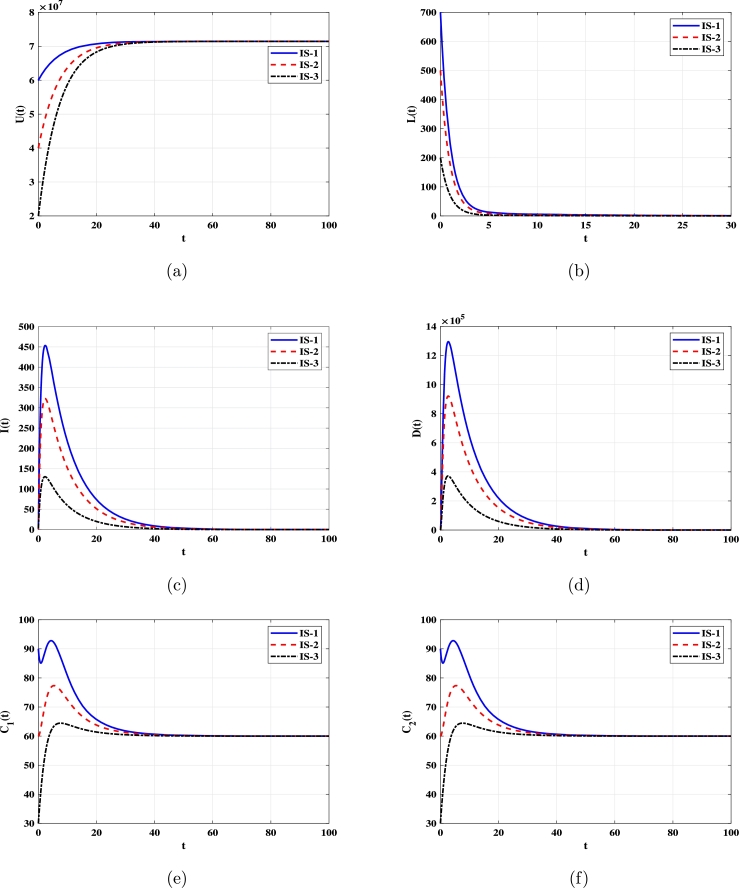
Solutions of system (22)-(27) with different initials converge to ${\mathrm{EQ}}^{0}=\left(7.143\times 10^{7},0,0,0,60,60\right)$ when $R_{0}^{L}\leq 1$. (a) Uninfected monocytes, (b) Latently infected monocytes. (c) Infected monocytes. (d) DENV. (e) Non-specific CTLs. (f) Strain-specific CTLs.

Various values of *α* are chosen under the given initial states, resulting in the following scenarios:

***Case 1 (Stability of***${\mathrm{EQ}}^{0}$***):*** Setting $\alpha =1.72\times 10^{-13}$, we obtain $R_{0}^{L}=0.23<1$. Figs. 5a–5f demonstrate that solutions with initials IS-1, IS-2, and IS-3 converge to the equilibrium ${\mathrm{EQ}}^{0}=\left(7.143\times 10^{7},0,0,0,60,60\right)$. This confirms that ${\mathrm{EQ}}^{0}$ is G.A.S according to Theorem 3. In this scenario, the counts of infected monocytes and DENV particles approach zero, while the number of uninfected monocytes returns to its normal value.

**Figure 6:**
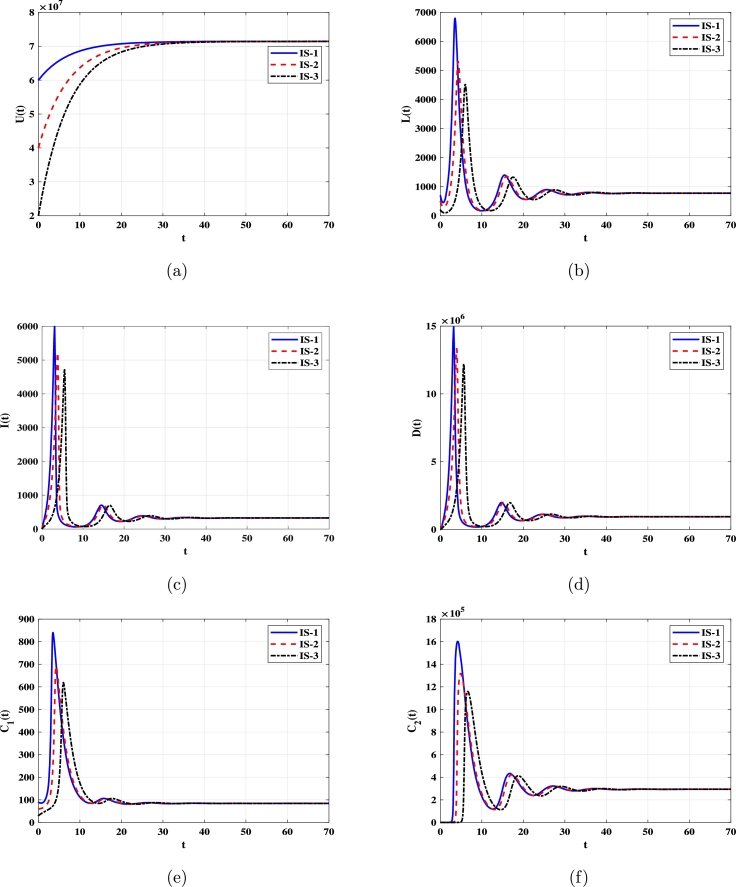
Solutions of system (22)-(27) with different initials converge to ${\mathrm{EQ}}^{⁎}=\left(7.142\times 10^{7},776.29,326.73,938881,84.52,293952\right)$ when $R_{0}^{L}>1$. (a) Uninfected monocytes, (b) Latently infected monocytes. (c) Infected monocytes. (d) DENV. (e) Non-specific CTLs. (f) Strain-specific CTLs.

***Case 2 (Stability of***${\mathrm{EQ}}^{⁎}$***)***: Choosing $\alpha =1.72\times 10^{-11}$ yields $R_{0}^{L}=22.7>1$. In Figs. 6a–6f, we observe that solutions starting with initials IS-1, IS-2, and IS-3 converge to ${\mathrm{EQ}}^{⁎}=\left(7.142\times 10^{7},776.29,326.73,938881,84.52,293952\right)$, and it is then G.A.S in accordance with Theorem 4.

### 4.3 Impact of CTL immune response and latently infected cells on the DENV dynamics

Now we discuss the impact of cytotoxic T lymphocyte (CTL) immune response and the presence of latently infected cells on the dynamics of DENV dynamics. If we neglect the CTL immune in the secondary DENV model, then we get

(40) $$\frac{dU}{dt}=\rho -\sigma U-\alpha UD,$$

(41) $$\frac{dI}{dt}=\alpha UD-\mu I,$$

(42) $$\frac{dD}{dt}=\eta I-\beta D.$$

The basic reproduction number of system (40)-(42) is defined as:

$${\overset{ˆ}{R}}_{0}=\frac{\eta \alpha \rho }{\mu \sigma \beta }.$$

Let us compare the basic reproduction numbers for system (6)-(10) and system (40)-(42) as:

$$R_{0}=\frac{\eta \alpha \rho }{\mu \sigma \beta \left(\frac{\kappa_{1}\varkappa_{1}}{{\text{ν}}_{1}\mu }+\frac{\kappa_{2}\varkappa_{2}}{{\text{ν}}_{2}\mu }+1\right)}=\frac{{\overset{ˆ}{R}}_{0}}{\frac{\kappa_{1}\varkappa_{1}}{{\text{ν}}_{1}\mu }+\frac{\kappa_{2}\varkappa_{2}}{{\text{ν}}_{2}\mu }+1}<{\overset{ˆ}{R}}_{0}.$$

Therefore, the presence of a CTL response diminishes the basic reproduction number, $R_{0}$, thereby enhancing the system's stabilizability around the uninfected equilibrium, ${\mathrm{EQ}}^{0}$. To assess the impact of integrating latently infected cells into the model, we compare the basic reproduction numbers for systems (6)-(10) and (22)-(27):

$$R_{0}^{L}=\frac{\eta \alpha \rho (\delta +\theta \lambda )}{\mu \sigma \beta (\delta +\lambda )\left(\frac{\kappa_{1}\varkappa_{1}}{{\text{ν}}_{1}\mu }+\frac{\kappa_{2}\varkappa_{2}}{{\text{ν}}_{2}\mu }+1\right)}=\frac{\delta +\theta \lambda }{\delta +\lambda }R_{0}<R_{0}.$$

This implies that incorporating latently infected cells results in a reduction of the basic reproduction number, leading to increased system stabilizability around the uninfected equilibrium ${\mathrm{EQ}}^{0}$.

## 5 Discussions and perspectives

In this paper, two secondary DENV infection models with non-specific and strain-specific CTLs were considered. The first model explains the interaction of five populations: uninfected monocytes, infected monocytes, free DENV particles, non-specific CTLs and strain-specific CTLs. Latently infected cells were included in the second model. We established the non-negativity and boundedness of solutions for the proposed model. Two equilibrium points emerged: the uninfected equilibrium, ${\mathrm{EQ}}^{0}$, and the infected equilibrium, ${\mathrm{EQ}}^{⁎}$, which depend on the basic reproduction number, $R_{0}$, determining the model's dynamic behavior. For $R_{0}\leq 1$, ${\mathrm{EQ}}^{0}$ is globally asymptotically stable (G.A.S), while for $R_{0}>1$, ${\mathrm{EQ}}^{⁎}$ exhibits G.A.S. Our theoretical findings were corroborated through numerical computations. Additionally, we conducted sensitivity analysis, examining how parameter values influence the basic reproduction number given a dataset. Furthermore, we examined the impact of incorporating CTL immune response and latently infected cells in the secondary DENV infection model, revealing a reduction in $R_{0}$ and an increased stabilizability around ${\mathrm{EQ}}^{0}$.

This paper has proposed a model where the in-host dynamics modeled at the small scale of particles is linked to the large scale of individuals. This multiscale modeling trend has been developed with a focus on COVID-19 epidemics, where the in-host dynamics determines the diffusion of infection in a population, see [34]. Therefore, we trust that the approach proposed in our work, after appropriate developments, can contribute to new trends in the study of epidemics. Moreover, our models can be improved by taking into account immunologic memory [35], [36].

## CRediT authorship contribution statement

**Aeshah A. Raezah:** Writing – original draft, Formal analysis. **A.M. Elaiw:** Writing – review & editing, Methodology, Investigation, Conceptualization. **M.A. Alshaikh:** Writing – original draft, Investigation, Formal analysis.

## Declaration of Competing Interest

The authors declare that they have no known competing financial interests or personal relationships that could have appeared to influence the work reported in this paper.

## Data Availability

No data was used for the research described in the article.
